# The prehistoric roots of Chinese cuisines: Mapping staple food systems of China, 6000 BC–220 AD

**DOI:** 10.1371/journal.pone.0240930

**Published:** 2020-11-04

**Authors:** Xinyi Liu, Rachel E. B. Reid

**Affiliations:** 1 Department of Anthropology, Washington University in St. Louis, St. Louis, Missouri, United States of America; 2 Department of Geosciences, Virginia Polytechnic Institute and State University, Blacksburg, Virginia, United States of America; University at Buffalo - The State University of New York, UNITED STATES

## Abstract

We conducted a meta-analysis of published carbon and nitrogen isotope data from archaeological human skeletal remains (n = 2448) from 128 sites cross China in order to investigate broad spatial and temporal patterns in the formation of staple cuisines. Between 6000–5000 cal BC we found evidence for an already distinct north versus south divide in the use of main crop staples (namely millet vs. a broad spectrum of C_3_ plant based diet including rice) that became more pronounced between 5000–2000 cal BC. We infer that this pattern can be understood as a difference in the spectrum of subsistence activities employed in the Loess Plateau and the Yangtze-Huai regions, which can be partly explained by differences in environmental conditions. We argue that regional differentiation in dietary tradition are not driven by differences in the conventional “stages” of shifting modes of subsistence (hunting-foraging-pastoralism-farming), but rather by myriad subsistence choices that combined and discarded modes in a number of innovative ways over thousands of years. The introduction of wheat and barley from southwestern Asia after 2000 cal BC resulted in the development of an additional east to west gradient in the degree of incorporation of the different staple products into human diets. Wheat and barley were rapidly adopted as staple foods in the Continental Interior contra the very gradual pace of adoption of these western crops in the Loess Plateau. While environmental and social factors likely contributed to their slow adoption, we explored local cooking practice as a third explanation; wheat and barley may have been more readily folded into grinding-and-baking cooking traditions than into steaming-and-boiling traditions. Changes in these culinary practices may have begun in the female sector of society.

## Introduction

Staple foods pass through a long transformative process as they are acquired, prepared, and distributed by human societies, and the performances of staple food preparation and presentation are intimately connected with social relationships [1]. Recent investigations have shown that between 5000 and 1500 cal BC, the Eurasian and African landmass underpinned a continental-scale process of food ‘globalisation’ of staple crops [2, 3]. By 1500 cal BC, the process brought together previously isolated agricultural systems to form a new kind of management system that enabled multi-cropping and fundamentally transformed Eurasian diets. China plays an important role in this narrative for its diverse forms of food products in the Neolithic but also as both the source of eastern domesticates (e.g., rice, broomcorn and foxtail millet) that moved from China to the West, and the recipient of southwest Asian grains (i.e. wheat and barley) that moved east-wards. Understanding the prehistoric roots of Chinese staple cuisines provides perspectives that not only transform our knowledge of the past but also raise awareness of the present and future utilities of these cereals.

Cultivation of staple cereals has played a vital role in the development of many aspects of Chinese culture from prehistory to today. Globally, the process employs millions of people and presently feeds 20% of the world’s population [4]. Cereals are the most important food source in the world, contributing as much as 70% of energy intake in developing countries [5]. In China, cereal foods such as rice and wheat products contributed 75–85% towards the daily dietary intake for average low/medium-income individuals in the 1980s [6]. Regional variations in cereal management and choice of staple products provide a key to understanding food production and consumption in China. These variations (e.g., rice in the lower Yangtze, wheat in the northwest, barley in Tibet) have been well documented historically [4, 7].

The diversity in staple choices has often been linked to origins of the diversity of regional cooking techniques. Early communities in East and West Asia, for example, were characterized by differences in food processing technologies: culinary traditions based on boiling and steaming of grain in the East, and by grinding grain and baking the resulting flour in the West. While the Pre-Pottery Neolithic cultures of Southwest Asia made extensive use of querns for flour production and constructed clay ovens for baking bread and roasting foods, cultures in Neolithic China elaborated forms of ceramic vessels for boiling, steaming, and serving [8]. Current evidence places pottery in south China *c*. 18,000 years ago, associated with hunter-gathers [13]. By contrast, in Southwest Asia, ceramics developed relatively late, dated *c*. 8,500 years ago [8]. This contrast has led to the hypothesis that these distinct East-West cooking technologies are deep seated in cultural differences between peoples that predate domestication [8]. In the context of early globalization of staple crops, the dispersal of cereals into new areas was not necessarily accompanied by the dispersal of culinary traditions. Novel grains could sometimes be incorporated into existing local traditions of food processing or sometimes lose the status of being staple grain.

Here, we integrate a large body of isotopic data from both English and Chinese publications to explore broad spatial and temporal patterns in the prehistoric roots of Chinese staple cuisines and assess possible gender distinctions in the context of staple consumption. Other recent reviews of this literature, though are not as broad in geographic scope, have shed important light on this topic [55, 9]. We additionally explore the nature of regional differences in staple traditions and consider the context in which culinary innovation arose.

**Fig 1:**
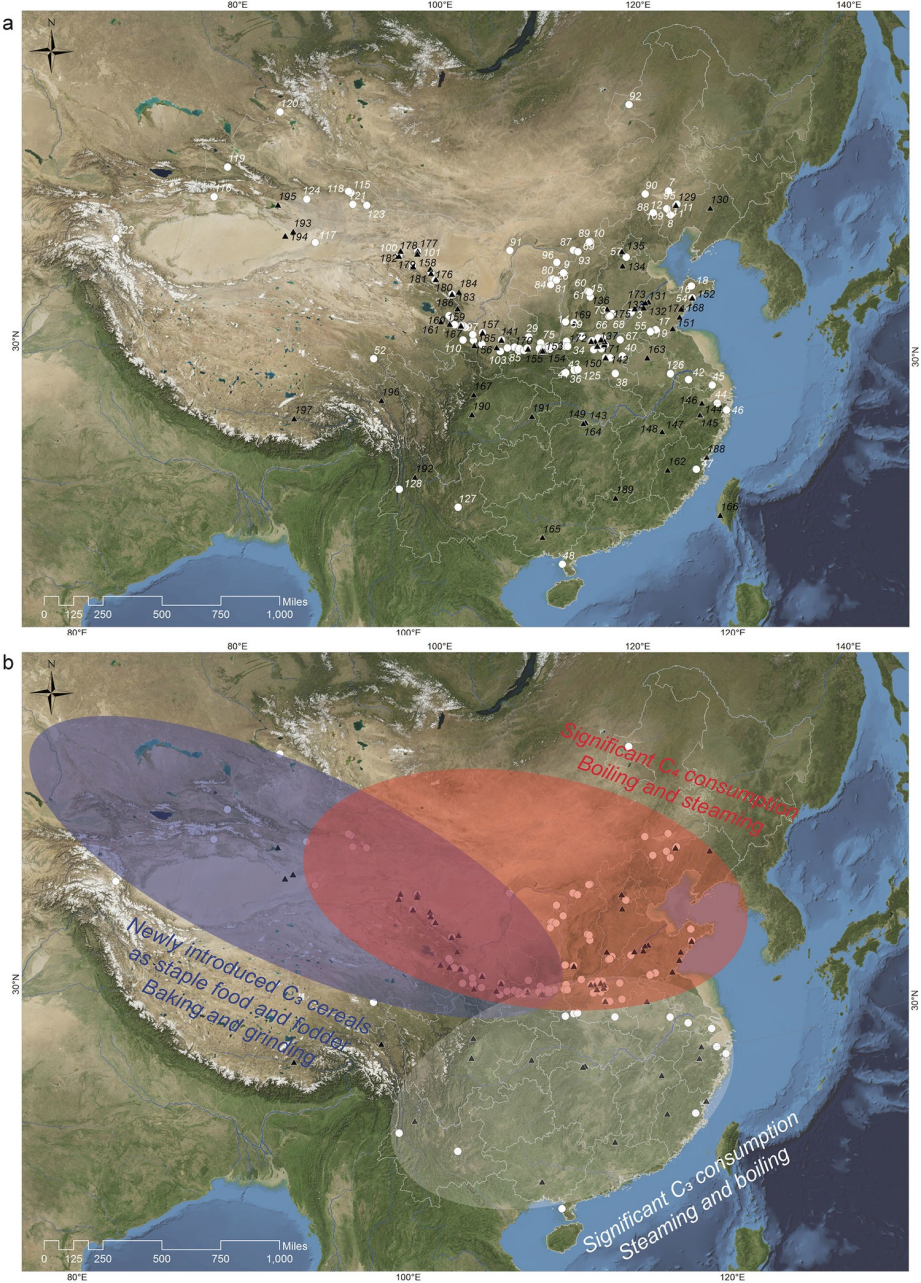
Site maps. (a) Site locations with isotope (white circles) and archaeobotanical (black triangles) data (see Tables 1–3, S1 for isotope studies and S2 for archaeobotanical). (b) Proposed culinary traditions in China after 2000 cal BC as described in the discussion. Regional difference in cooking follows the hypothesis proposed by Fuller and Rowlands (2011). Map generated using ArcMap v. 10.2 and NASA Blue Marble with data set obtained from NASA Earth Observatory (public domain). See: http://earthobservatory.nasa.gov/Features/BlueMarble/.

Isotopic values from archaeological skeletons provide direct proxies for long-term consumption practices of individuals in the past. Due to the resolution of this technique–which doesn’t enable assessment of the contribution of minor dietary components–we focus our discussion on the consumption of staple foods. Carbon isotope values (*δ*^13^C) vary primarily according to the photosynthetic pathways employed by plants at the base of the food chain [10, 11]. The potential for using δ^13^C values to differentiate between different types of cereal diets was first realized in detecting the C_4_ domesticate maize (*Zea mays*) in Americas [10]. Nitrogen isotope values (*δ*^15^N) provide further information about past diets by situating the consumers on the trophic food chain; *δ*^15^N values increase by 3–5 ‰ with each step in a trophic chain [12]. Nonetheless, without site specific faunal baseline data, dietary reconstruction with *δ*^15^N values at this broad geographic scope is challenging. There are now over 90 publications presenting isotopic results from >120 sites and >2000 human individuals from prehistoric China. We compiled these data to investigate the historic geography of staple cuisines between 6000 cal BC and 220 cal AD, capitalizing on the contrasting isotopic signatures among major crop domesticates, including rice, wheat and barley (C_3_ plants), and broomcorn and foxtail millets (C_4_ plants). We focus on three regions featuring differing environmental characteristics and distinct agricultural and culinary traditions: (1) the broad Loess Plateau including the Yellow, Wei and Xiliao Rivers, (2) the Yangtze and Huai Rivers, and (3) the Continental Interior bordering the Loess Plateau and Eurasian steppe including the Tibetan Plateau (Fig 1).

### Setting up the geography

China’s vast landmass ranges across contrasting ecological extremes, from tropical in the south, to sub-Arctic in the north, alpine in the west and marshy lowlands in the east [13]. A key dynamic climatic element is the monsoonal system, comprising a warm, wet summer monsoon, and a cold, dry winter monsoon. The summer monsoon brings water from the Pacific and Indian Oceans onto much of the east and south of China while the winter monsoon drives the movement of Aeolian dust from the Gobi desert to the Loess Plateau. The sensitivity of the monsoonal system to fluctuations in the relative temperatures of land and ocean has rendered it the most variable part of the physical environment, critically affecting water availability in many parts of China, particularly towards the south and east [13]. These features have led to an agriculture that is diverse in its crop ecology, elaborate in its management of water, and with its most intense sedentary cultivations in the east of the country, including the broad Loess Plateau and the Yangtze and Huai Rivers, which is geographically divided by the Qinling Mountains and Huai River [14, 15].

The central/eastern parts of China host the most productive soils and have an enduring association with important staple cereals: the Yangtze and Huai Rivers with rice (*Oryza sativa*), and the Loess Plateau with broomcorn and foxtail millet (*Panicum miliaceum* and *Seteria italica*) [13, 16]. The oldest archaeological sites preserving broomcorn and foxtail millet remains do not, however, lie in direct proximity to the great rivers. Sites with millet remains are instead located along the foothills of the eastern edge of the Loess Plateau at a considerable distance from the rivers themselves [17]. The earliest sites with rice are situated in the middle and lower Yangtze and Huai River valleys [18] at locations associated with minor tributaries and inter-mountain plains where cultivation could be easily managed [19]. In the archaeobotanical record dating to before 5000 cal. BC, a north-south divide is observable on either side of the Huai River–Qinling Mountain line, a topographical reference used by modern geographers to distinguish between north and south China. North of this line, millet cultivation was predominant in the Loess Plateau, while south of the line, subsistence was based on a diverse spectrum of food resources including cultivation of rice and managing free-living plants prevalent in the Yangtze-Huai Region [15, 16].

The same general area of central/eastern China came into contact with Central Asia and possibly South Asia in the Bronze Age, facilitating the adoption of a variety of cereal crops originating in the west [2]. To the west and north of this principal area of Chinese agriculture, the Loess Plateau and the upper Yangtze is flanked by the mountainous Continental Interior. This includes the Mongolian plateau, the Gobi desert, the Hexi Corridor, western Sichuan and northern Yunnan bordering the eastern Tibetan Plateau, as well as the northern and eastern parts of the Tibetan Plateau itself. In the context of a trans-Eurasian exchange of cereal crops, the founder crops from the Fertile Crescent (modern-day Iran, Iraq, Syria and southern Turkey)–notably free-threshing wheat (*Triticum aestivum*) and naked barley (*Hordeum vulgare*)–were introduced to China between 3000 and 1500 cal BC possibly along multiple routes through the Continental Interior [20]. The introduction of the Fertile Crescent grains in the Bronze Age significantly transformed the staple food system in China.

Prehistoric people did not subsist on cereals alone. Archaeobotanical evidence shows that, since the terminal Pleistocene, a variety of plants including acorns, beans, tubers and grasses (*Triticeae* and *Paniceae*) were used in the Loess Plateau [21]. Over the course of the Neolithic (c. 8000–1500 BC), additional plant and animal domesticates were introduced into human and animal diets, including pigs (*Sus scrofa*), soybean (*Glycine max*), adzuki bean (*Vigna angularis*), buckwheat (*Fagopyrum esculentum*), and hemp seed (*Cannabis sativa*) [13, 22]. Recent research shows that in the Yangtze-Huai region, rice cultivation emerged in the context of broad spectrum foraging focused on the collection of tree nuts, especially acorns (*Quercus* spp.), fruits such as peaches and apricots (*Prunus* spp.), and wetland nuts and tubers, including water chestnuts (*Trapa natans*), foxnuts (*Euryale ferox*), lotus root (*Nelumbo nucifera*), job’s tears (*Coix lachrymal-jobi*) and barnyard grasses (*Echinochloa* spp.) [23–25]. With the exception of jobs’ tears and some members of *Triticeae* and *Paniceae*, all the fruits, nuts, tubers and beans identified employ the C_3_ photosynthetic pathway.

## Materials and methods

To locate published archaeological isotopic studies from China, we searched Web of Science and Google Scholar using combinations of the following keywords: stable isotopes, China, human diet, bone collagen, and apatite. To include data published in Chinese, we searched the China Academic Journals Database using the same set of keywords. We restricted our search to articles concerned with post-Paleolithic archaeological sites and specimens dating to before 220 AD, the ending point of the Han Dynasty. Our search yielded isotopic data from 128 sites in over 90 articles published in English and Chinese between 1984 and 2018 (Tables 1–3, S1 Table). The articles are primarily concerned with *δ*^13^C and *δ*^15^N values from archaeological human bone collagen (n = 83, including 7 review articles), although a subset includes carbon and oxygen isotope data (*δ* ^13^C and *δ* ^18^O) from bone apatite and/or tooth enamel (n = 6). Several recent articles also feature sulphur isotope data (*δ* ^34^S), but these are presently few in numbers (n = 6). We did not consider articles focusing on strontium (Sr) isotope ratios, which are commonly used as a geographical fingerprinting tool. As we only use published data for the meta-analysis, no permits were required for this study, which compiled with all relevant regulations. All published data compiled in this study is presented in S1 Table and summarized in Tables 1–3 with references to the original studies. Specimen IDs (where are available) as given in the original isotopic studies are also presented in S1 Table.

**Table 1:** Published human C and N isotope values from before 5000 cal BC.

Site	Site number	Region	Province	Cultural group	Estimated dates (cal. BC/AD)	Mean δ^15^N	SD δ^15^N	Mean δ^13^C	SD δ^13^C	n	Reference number
Xinglonggou	1	A	Inner Mongolia	Xinglongwa	6200–5300	9.8	0.8	-9.9	1.1	30	[43, 59]
Xinglongwa	2	A	Inner Mongolia	Xinglongwa	6200–5300	-	-	-8.9	1.7	7	[59]
Xiaojingshan	3	A	Shandong	Houli	6200–5500	9.0	0.6	-17.8	0.3	10	[60]
Baijia	4	A	Shaanxi	Laoguantai	5700–5300	10.9	1.7	-13.7	1.9	3	[61]
Beiliu	5	A	Shaanxi	Laoguantai	6000–5000	8.5	0.1	-12.0	0.6	6	[62, 63]
Jiahu	6	B	Henan	Jiahu	7000–5800	8.9	0.9	-20.3	0.5	14	[64]

Summary of published carbon and nitrogen isotope values measured in human bone collagen from sites dating to before 5000 cal BC.

**Table 2:** Published human C and N isotope values from between 5000 and 2000 cal BC.

Site	Site number	Region	Province	Cultural group	Estimated dates (cal. BC/AD)	Mean δ^15^N	SD δ^15^N	Mean δ^13^C	SD δ^13^C	n	Reference number
Baiyinchanghan	7	A	Inner Mongolia	Hongshan	4300–3900	8.6	0.3	-8.8	0.4	3	[43]
Caomaoshan	8	A	Inner Mongolia	Hongshan	3400–3100	9.1	0.4	-9.3	0.6	7	[43]
Dakou	9	A	Inner Mongolia	Dakou	2300–1900	7.5	1.0	-8.9	1.8	2	[65]
Miaozigou	10	A	Inner Mongolia	Miaozigou	3500–3000	9.2	0.2	-7.2	0.2	9	[66]
Xinglongwa	11	A	Inner Mongolia	Hongshan	4500–3000	8.7	-	-5.4	-	1	[59]
Xishan	12	A	Inner Mongolia	Xiaoheyan	2900–2400	8.8	0.4	-7.5	0.5	16	[42]
Qingliangsi	13	A	Shanxi	Yangshao/Longshan	5000–2000	8.5	1.1	-8.1	0.9	27	[67]
Taosi	14	A	Shanxi	Longshan	2500–2000	8.9	1.3	-7.3	2.4	15	[68, 69]
Xinhuacun	15	A	Shanxi	Longshan	2300–2000	6.5	1.4	-7.3	0.7	2	[65]
Beiqian	16	A	Shandong	Dawenkou	4000–3000	8.8	1.0	-9.6	0.8	38	[44]
Beizhuang	17	A	Shandong	Beizhuang	4500–2500	13.2	-	-7.9	-	1	[59]
Guzhendu	18	A	Shandong	Dawenkou	4000–3000	9.6	-	-8.5	0.7	4	[59]
Xigongqiao	19	A	Shandong	Dawenkou	4000–3000	8.1	2.1	-15.3	3.8	10	[70]
Banpo	20	A	Shaanxi	Yangshao	4800–4300	9.1	-	-15.0	-	1	[42]
Beiliu	21	A	Shaanxi	Yangshao	4000–3500	8.7	0.9	-12.0	1.4	6	[62, 63]
Beishouling	22	A	Shaanxi	Yangshao	5000–3500	-	-	-13.8	0.9	3	[68]
Dongying II	23	A	Shaanxi	Longshan	2600–2000	9.4	0.3	-8.0	1.3	5	[71]
Jiangzhai	24	A	Shaanxi	Yangshao	4900–4000	8.6	0.6	-9.9	1.2	20	[42, 72]
Quanhucun	25	A	Shaanxi	Yangshao	3500–3000	11.5	-	-11.2	-	1	[73]
Shengedaliang	26	A	Shaanxi	Longshan	2500–2000	8.8	1.4	-8.5	1.8	28	[74]
Shijia	27	A	Shaanxi	Yangshao	4300–4000	8.1	0.5	-10.0	0.7	9	[42]
Xipo	28	A	Shaanxi	Yangshao	4000–3300	9.4	1.0	-9.7	1.1	31	[75]
Xunyi	29	A	Shaanxi	Longshan	2400–2000	8.2	0.1	-7.1	0.1	3	[65]
Yuhuazhai	30	A	Shaanxi	Yangshao	5000–3000	8.4	1.9	-10.9	4.4	35	[65, 75]
Zhouyuan	31	A	Shaanxi	Longshan	2500–2000	6.7	3.9	-8.0	0.7	5	[76]
Guanjia	32	A	Henan	Yangshao	4000–3500	6.2	0.7	-8.0	0.6	21	[50]
Wadian	33	A	Henan	Wangwan III	2400–2000	8.2	1.3	-11.0	2.1	12	[77]
Xiaowu	34	A	Henan	Yangshao	5000–4000	7.8	0.8	-10.3	1.2	74	[78]
Xishan	35	A	Henan	Yangshao	5000–3000	9.0	0.8	-8.2	1.5	39	[75]
Gouwan	36	B	Henan	Yangshao/Qujialing	5000–2600	8.3	1.1	-14.3	1.9	41	[79]
Haojiatai	37	B	Henan	Longshan	2500–2000	9.2	1.1	-13.1	4.9	11	[130]
Jiazhuang	38	B	Henan	Longshan	2500–2000	12.7	-	-19.1	-	1	[130]
Meishan	39	B	Henan	Longshan	2500–2000	10.2	1.5	-15.0	2.8	4	[130]
Pingliangtai	40	B	Henan	Longshan	2500–2000	9.0	1.0	-8.7	1.2	8	[130]
Xiazhai	41	B	Henan	Longshan	2500–2000	8.2	0.7	-10.2	1.9	22	[130]
Sanxingcun	42	B	Jiangsu	Majiabang	4500–3500	9.7	0.3	-20.0	0.2	19	[81]
Qinglongquan	43	B	Hubei	Qujialing/Shijiahe	3000–2200	9.0	1.1	-14.6	1.4	27	[82, 83]
Hemudu	44	B	Zhejiang	Hemudu	5000–4000	11.4	0.3	-18.2	2.2	4	[59]
Songze	45	B	Zhejiang	Songze	4000–3300	10.9	1.6	-19.9	0.4	2	[59]
Tashan	46	B	Zhejiang	Hemudu-Majiabang	3900–2200	6.9	4.6	-20.7	4.5	4	[84]
Tanshishan	47	B	Fujian	Tanshishan	3000–4300	10.8	1.6	-18.4	1.1	17	[46]
Liyudun	48	B	Guangdong		c. 5000	13.8	1.4	-17.0	1.3	2	[44]
Hupo	49	C	Qinghai	Banshan/Machang	2300–2000	7.6	0.3	-8.7	0.4	6	[85]
Mozuizi	50	C	Gansu	Machang	2400–2000	8.3	0.4	-7.2	0.4	14	[47]
Wuba	51	C	Gansu	Banshan/Machang	2500–1900	9.2	1.1	-7.5	1.3	55	[47]
Zongri	52	C	Qinghai	Zongri	3700–2300	8.3	0.5	-10.1	1.1	24	[86]
Dadiwan	53	C	Gansu	Yangshao	4550–2950	9.7	0.8	-9.8	3.0	6	[87]

Summary of published carbon and nitrogen isotope values measured in human bone collagen from sites dating to between 5000 and 2000 cal BC.

**Table 3:** Published human C and N isotope values from between 2000 cal BC and 212 cal AD.

Site	Site number	Region	Province	Cultural group	Estimated dates (cal. BC/AD)	Mean δ^15^N	SD δ^15^N	Mean δ^13^C	SD δ^13^C	n	Reference number
Beiqian	54	A	Shandong	Zhou Dynasty	1046–256	10.5	0.5	-9.2	0.8	4	[44]
Qianzhangda	55	A	Shandong	Shang/Western Zhou	1200–771	10.0	1.2	-8.9	1.4	49	[88]
Xiyasi	56	A	Shandong	Eastern Zhou	770–221	8.1	0.9	-11.9	2.2	30	[50]
Liulihe	57	A	Hebei	Western Zhou	1046–771	-	-	-8.2	1.4	19	[59]
Nancheng	58	A	Hebei	Proto Shang	2000–1600	9.4	0.0	-6.9	0.1	75	[89]
Neiyangyuan	59	A	Shanxi	Spring and Autumn	770–476	9.6	1.0	-8.6	1.6	23	[90]
Niedian	60	A	Shanxi	Xia	1900–1500	10.5	0.7	-7.1	0.3	60	[91]
Xiaonanzhuang	61	A	Shanxi	Eastern Zhou	770–221	10.5	0.9	-8.0	0.4	16	[92]
Xiaoshuangqiao	62	A	Shanxi	Shang	1600–1046	8.5	1.6	-10.0	1.7	10	[93]
Anyang	63	A	Henan	Shang	1400–1046	-	-	-8.2	2.5	39	[59]
Changxinyua	64	A	Henan	Eastern Zhou	770–221	7.7	1.0	-10.3	1.4	15	[50]
Erlitou	65	A	Henan	Erlitou	1900–1500	11.9	4.2	-9.4	2.1	31	[59, 69]
Handeng	66	A	Henan	Proto Shang	1750–1600	9.3	-	-6.7	-	1	[94]
Liuzhuang	67	A	Henan	Proto Shang	2000–1600	9.7	1.5	-8.2	1.9	21	[95]
Xiaomintun	68	A	Henan	Shang	1400–1046	9.5	0.6	-11.5	2.7	4	[96]
Xinzhai	69	A	Henan	Erlitou	1900–1500	9.0	1.0	-9.6	1.4	8	[97]
Xinzheng City	70	A	Henan	Eastern Zhou	770–221	8.8	0.8	-11.0	1.6	75	[80]
Xuecun	71	A	Henan	Han	202BC-220AD	10.6	1.3	-13.7	1.2	53	[80]
Yanshi	72	A	Henan	Shang	1600–1046	-	-	-7.6	0.8	3	[59]
Yinxu	73	A	Henan	Shang	1250–1046	9.1	1.2	-8.5	1.0	130	[98–100, 104]
Fenggeling	74	A	Shaanxi		500–300	9.1	0.4	-9.2	0.5	4	[65]
Guandao	75	A	Shaanxi	Han	202BC-220AD	10.4	0.3	-10.7	0.8	5	[101]
Guangming	76	A	Shaanxi	Western Han	202BC-8AD	11.0	0.8	-9.8	0.9	7	[101]
Jianhe	77	A	Shaanxi	Warring States	476–221	8.7	0.5	-9.2	0.7	14	[102]
Jichang	78	A	Shaanxi	Eastern Han	8-220AD	9.0	0.9	-12.0	1.2	30	[101]
Lintong	79	A	Shaanxi		300BC-700AD	9.4	1.3	-13.8	3.8	3	[65]
Muzhuzhuliang	80	A	Shaanxi	Longshan/Xia	1900–1700	8.8	0.6	-8.2	1.5	8	[103]
Shimao	81	A	Shaanxi	Longshan/Xia	2100–1600	6.9	0.9	-8.4	0.1	4	[65]
Shigushan	82	A	Shaanxi	Western Zhou	1200–900	9.4		-9.8		1	[104]
Sunjianantou	83	A	Shaanxi	Eastern Zhou	770–221	8.5	1.0	-10.8	1.3	25	[105]
Xinhua	84	A	Shaanxi		2000–1700	8.2	-	-8.7	-	1	[65]
Zhanguo	85	A	Shaanxi		450–350	8.8	-	-14.8	-	1	[65]
Zhouyuan	86	A	Shaanxi	Western Zhou	1200–900	9.8	1.3	-10.9	2.3	9	[104]
Dabaoshan	87	A	In. Mongolia	Warring States	410–180	9.6	0.9	-9.0	1.4	40	[106]
Dashanqian	88	A	In. Mongolia	Upper Xiajiadian	900–200	9.3	0.6	-7.0	0.4	9	[107]
Huhewusu	89	A	In. Mongolia	Western Han	202BC-8AD	9.1	0.6	-9.1	0.7	5	[108]
Jinggouzi	90	A	In. Mongolia	Eastern Zhou	770–221	9.8	0.6	-12.4	0.7	10	[109]
Nalintaohai	91	A	In. Mongolia	Western Han	202BC-8AD	13.3	1.2	-10.0	0.8	7	[110]
Tuoba Xianbei	92	A	In. Mongolia	Tuoba Xianbei	100BC-557AD	10.4	1.3	-11.4	2.8	65	[111]
Xindianzi	93	A	In. Mongolia	Eastern Zhou	770–221	10.3	0.8	-11.5	0.9	20	[112]
Xinglongwa	94	A	In. Mongolia	Lower Xiajiadian	1500–1300	-	-	-4.2	0.9	2	[59]
Xinglongwa III	95	A	In. Mongolia	Lower Xiajiadian	1500–1300	9.8	0.9	-7.0	0.6	9	[43, 59]
Zhukaigou	96	A	In. Mongolia	Zhukaigou	2200–1600	9.0	1.1	-8.2	0.4	2	[65]
Buziping	97	C	Gansu	Qijia	2100–1700	8.1	-	-7.3	-	1	[48]
Buzishan	98	C	Gansu	Qijia	2100–1700	8.3	-	-7.3	-	1	[48]
Ganguai	99	C	Gansu	Siba	1400–900	11.6	0.9	-15.3	1.5	30	[83]
Huoshaogou	100	C	Gansu	Siba	2000–1300	12.0	1.3	-12.0	1.9	30	[83]
Huoshiliang	101	C	Gansu		2135–1690	8.0	2.6	-8.8	0.1	2	[58]
Lianhuatai	102	C	Gansu	Xindian	1500–1000	8.6	0.3	-10.0	0.3	6	[104]
Lixian	103	C	Gansu	Spring and Autumn	750–500	8.8	0.3	-13.1	4.1	3	[62]
Mogou	104	C	Gansu	Qijia/Siwa	1800–1100	10.2	1.2	-13.9	1.5	85	[83, 87]
Mozuizi	105	C	Gansu	Han	202BC-220AD	10.5	0.8	-15.7	1.4	6	[83]
Qijiaping	106	C	Gansu	Qijia	1500–1200	9.8	0.9	-8.9	1.1	42	[111]
Xiahaishi	107	C	Gansu	Qijia	2200–1900	8.6	1.0	-7.5	0.3	13	[48, 104]
Xichengyi	108	C	Gansu	Siba	2000–1000	11.7	2.1	-9.0	0.6	4	[113]
Xishan	109	C	Gansu	Spring and Autumn	770–403	7.9	1.8	-13.4	4.0	41	[85]
Zhanqi	110	C	Gansu	Siwa	1100–900	10.4	0.6	-15.5	1.0	31	[47, 104]
Lajia	111	C	Qinghai	Qijia	2000–1200	10.0	0.2	-7.9	0.4	4	[114]
Lajigai	112	C	Qinghai	Kayue	1400–1000	9.0	0.5	-14.9	1.8	5	[85]
Sanheyi	113	C	Qinghai	Qijia	2000–1800	8.1	1.5	-9.1	0.5	5	[85]
Shangsunjia	114	C	Qinghai	Kayue	1500–600	9.8	1.4	-16.2	1.3	21	[59]
Donghuigou	115	C	Xinjiang	Hongshankou	900–0	13.3	0.6	-18.4	0.4	13	[115]
Duogang	116	C	Xinjiang	Qunbake	900–500	12.6	0.6	-14.5	1.0	39	[116]
Gumugou	117	C	Xinjiang	Xiaohe	c. 1800	14.6	0.6	-18.2	0.2	10	[117, 118]
Heigouliang	118	C	Xinjiang	Western Han	500BC-8AD	12.5	0.6	-18.5	0.4	36	[119, 120]
Qiongkeke	119	C	Xinjiang	Early Iron Age	500–202	12.7	0.4	-16.2	0.2	8	[121]
Kelasu	120	C	Xinjiang	Early Iron Age/Han	500BC-220AD	11.8	0.6	-16.6	0.4	7	[122]
Tianshanbeilu	121	C	Xinjiang	Tianshanbeilu	2000–1300	14.7	0.9	-15.4	1.3	124	[123, 124]
Xiabandi	122	C	Xinjiang	Andronovo	1500–600	12.3	1.0	-18.2	0.8	27	[125]
Yanbulake	123	C	Xinjiang	Early Iron Age	1000–500	-	-	-14.6	1.7	2	[59]
Yanghai	124	C	Xinjiang		1200–100	12.1	1.0	-16.3	1.1	22	[126]
Shenmingpu	125	B	Henan	Warring States/Han	475BC-220AD	8.5	1.1	-14.6	2.2	32	[94]
Boyangcheng	126	B	Anhui	Spring and Autumn	1122–771	10.9	1.0	-18.8	1.5	38	[127]
Jinlianshan	127	B	Yunnan		700	9.8	0.9	-18.8	0.4	9	[128]
Shilinggang	128	B	Yunnan		850–250	10.0	1.0	-18.8	0.7	16	[129]

Summary of published carbon and nitrogen isotope values measured in human bone collagen from sites dating to between 2000 cal BC and 212 cal AD.

We performed all statistical analyses in R [26]. Before beginning analyses, we filtered out samples with poor C:N ratios (< 2.9 or > 3.6) suggesting that they were contaminated or poorly preserved. We described the basic structure of the data by calculating the mean and standard deviations of the isotope data by time period, region, province and/or sex. We recognize that present-day provinces are artificial borders, however for the sake of simplicity, we compare isotopic data among provinces as a way to examine north to south and east to west geographic gradients. Although these data did not always conform to the assumptions of parametric statistics (specifically, residuals were not always normally distributed and groups did not necessarily have equal variance), we nonetheless chose to use ANOVA with posthoc Tukey’s test for multigroup comparisons and were cautious about rejecting the null-hypothesis when *p*-values were close to 0.05. In the case of highly heteroscedastic groups, we used the more conservative Welch’s ANOVA for multi-group comparisons (see S3A–S3D Table and S4A–S4H Table for results).

### Mixing model

To estimate the proportional contributions of potential plant and animal food resources to past human diets at Xinglonggou—one of the oldest sites at which humans were using millet as a staple food—we used the Bayesian stable isotope mixing model MixSIAR [27, 28] following the best practices for stable isotope mixing models outlined by Phillips et al. [29]. We grouped dietary items into ecologically relevant isotopically distinct source groups by assessing whether sources had significantly different means using MANOVA followed by Tukey tests; dietary items that were isotopically indistinct (*p* > 0.05) were grouped and averaged over all of the samples. We accounted for concentration dependence by including the digestible [C] and [N] of the potential dietary resources, which we calculated from data available in the United States Department of Agriculture (USDA) Nutrient Database following Koch and Phillips [30]. To account for human diet-to-collagen isotope discrimination, we used a nitrogen isotope dietary discrimination factor of 3.5 ± 1 ‰ and a carbon isotope discrimination factor of 5 ± 1 ‰ [12, 31]. We initially tried nitrogen isotope discrimination factors of between 4.6–6 ‰ [32], but found that these values placed the human collagen samples well outside the dietary mixing space. To evaluate the sensitivity of the model to the nitrogen isotope discrimination factor, we ran the model using several discrimination factors (S5 Table); the estimated mean proportional contribution of C_4_ plants to human diet varies by just 4% among the models. We conducted Markov Chain Monte Carlo (MCMC) sampling within MixSIAR using the “very long” chain length, which includes running three replicate chains, each with 1,000,000 draws, a burn-in of 500,000, and a thinning rate of 500. We used Gelman-Rubin diagnostics to confirm model convergence [33]. Although the relative abundance of various dietary resources found at the archaeological sites could arguably be used to construct informative priors, we chose to use uninformative priors (i.e., flat) for past human diet because of the potential for differences in preservation and/or sampling effort between floral and faunal material.

### Kellner and Schoeninger’s approach

Partitioning the relative contributions of plant and animal resources to human diet is difficult to accomplish using stable isotope values of bulk collagen alone because collagen *δ*^13^C and *δ*^15^N values reflect both dietary protein and dietary non-protein disproportionately (approximately 60% of the carbon atoms in collagen come from dietary protein [10, 34–38]. One approach to addressing this issue is to use *δ*^13^C values in collagen and apatite from the same individual to model the regression lines of energy and protein sources, as collagen and bioapatite reflect dietary protein and the whole diet disproportionately [39]. Using the limited available data, we additionally followed [39] approach of plotting collagen *δ*^13^C against apatite *δ*^13^C with their modern-calibrated C_3_ and C_4_ protein regression lines [40, 41]. We identified three populations between 5000–2000 cal BC to be included in the analysis: Jiangzhai, Shijia and Banpo [42]. We also included Jiahu, a site that predates 5000 cal BC.

## Results

### Mapping staple suisines in prehistoric China

We considered temporal and spatial patterns by organizing the results in three successive periods: 6000–5000 cal BC, 5000–2000 cal BC, and post 2000 cal BC (Liu et al. 2019), and within the geographic framework of the three regions described above: the broad Loess Plateau, the Yangtze-Huai Region, and the Continental Interior (Fig 1).

#### 6000–5000 cal BC

**Fig 2:**
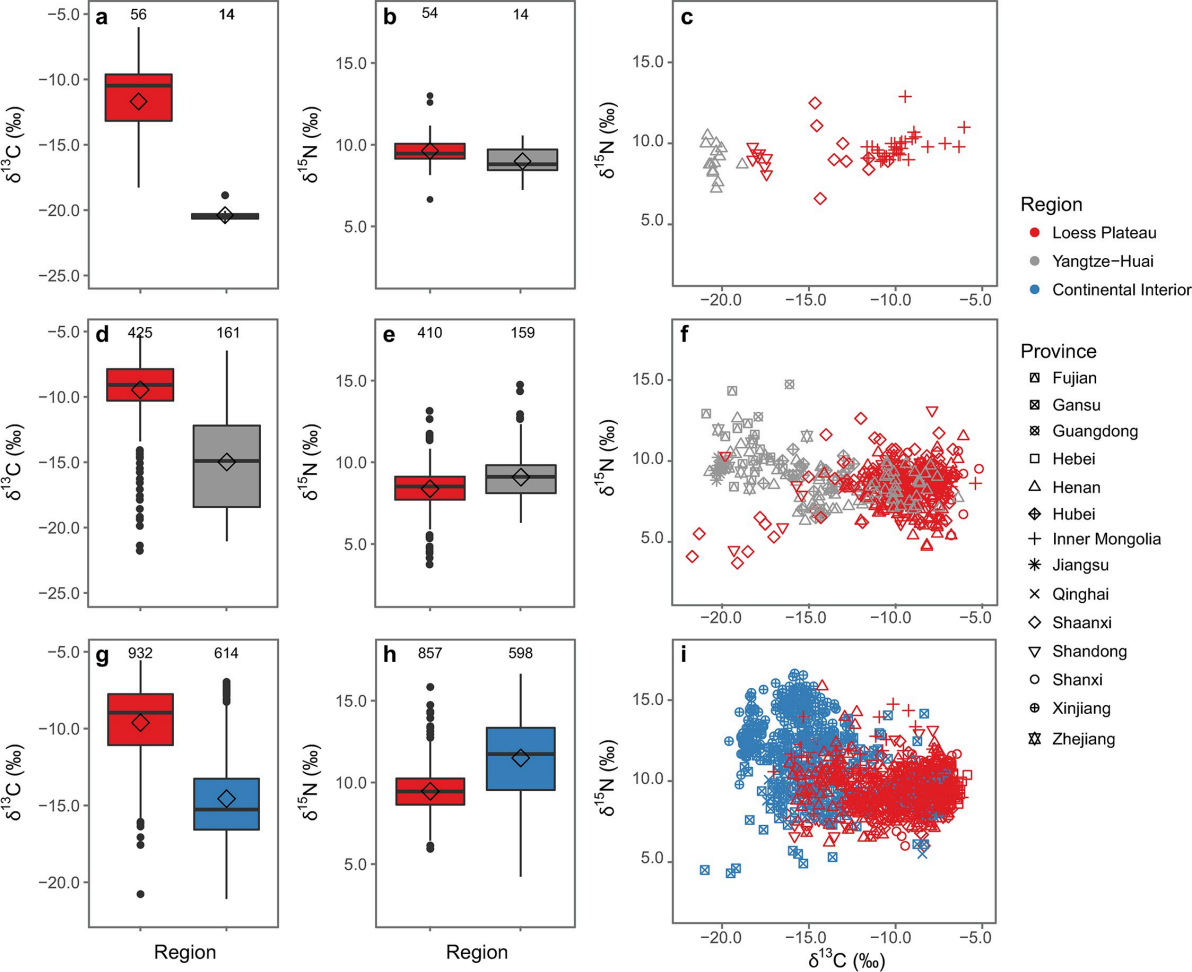
Box- and scatterplots of *δ*^13^C and *δ*^15^N values measured in human bone collagen. Data are from sites occupied pre-5000 cal BC (a-c), 5000–2000 cal BC (d-f), and post-2000 cal BC (g-i). Regions are differentiated by color and provinces by shape. Boxplots illustrate minimum, first quartile, median, third quartile, and maximum; means are depicted as hollow black diamonds and outliers as black dots. See Tables 1–3 for data citations and S1 Table for original data.

Carbon and nitrogen isotope values measured in human bones are reported from five sites dating to the period between 6000–5000 cal BC (Fig 2A–2C). With the exception of Jiahu from the Yangtze-Huai Region, the sites are located in the Loess Plateau. Carbon isotope ratios from Jiahu are consistent with a predominantly-C_3_ diet (mean *δ*^13^C < -17‰). In the Loess Plateau, human values from Xiaojingshan, Baijia and Beiliu are consistent with a mixed C_3_-C_4_ diet (*δ*^13^C values from -17 to -12‰), while at Xinglonggou and Xinglongwa, people have carbon isotope values indicative of a C_4_-plant dominated diet (mean *δ*^13^C > -12‰). The two regions have significantly different *δ*^13^C and *δ*^15^N values (*δ*^13^C: F_1,68_ = 93.4, *p* = 2.16 e-14, *δ*^15^N: F_1,66_ = 4.8, *p* = 0.0319).

**Fig 3:**
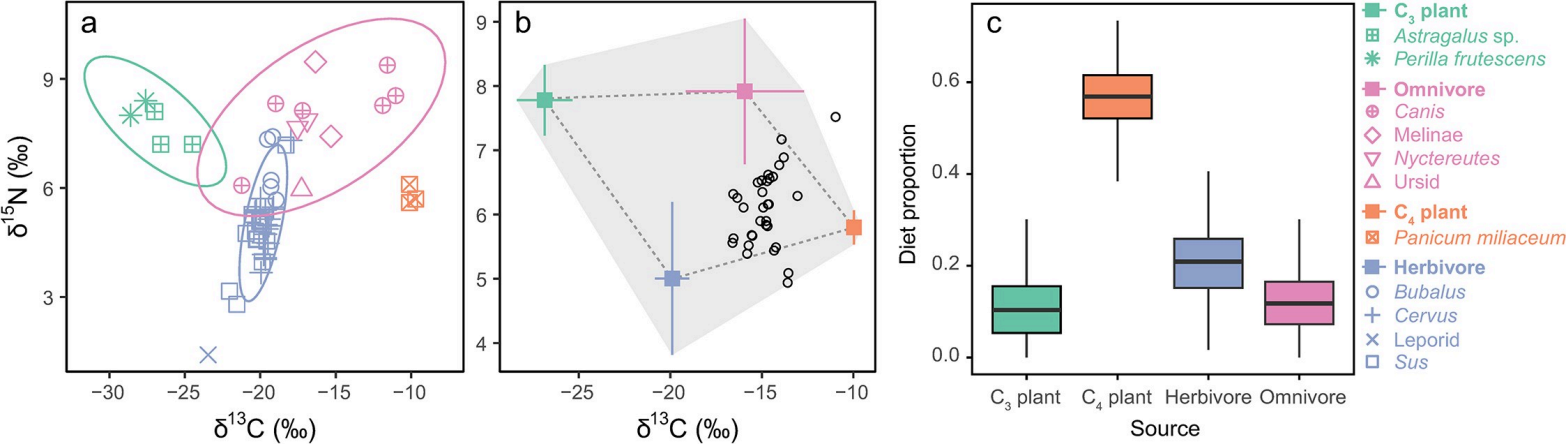
Xinglonggou mixing model results. (a) Animal bone collagen and plant *δ*^13^C and *δ*^15^N values from Xinglonggou; standard ellipse areas are depicted as ovals (C_3_ plant—green, omnivore—pink, C_4_ plant—orange, and herbivore—purple). (b) *δ*^13^C and *δ*^15^N values for grouped dietary sources (mean ± 1 std dev) plotted with trophic corrected *δ*^13^C and *δ*^15^N values from human bone collagen from Xinglonggou. The dashed line and the gray area illustrate the minimum and maximum convex hulls for the dietary mixing space, respectively. And (c) median (*lines in center of boxes* = median, *box boundaries* = 50% CI, *error bars* = 50% CI) proportional contributions of each dietary resource to humans from Xinglonggou.

Xinglonggou I (c. 6000 cal BC) provides a unique case study, as there are additionally data available from a range of both plant and animal dietary sources. At Xinglonggou, *δ*^13^C values measured in human bone collagen are consistent with a C_4_ diet with relatively high *δ*^15^N values (Fig 3B; mean *δ*^13^C = -9.9 ± 1.1 ‰; *δ*^15^N = 9.8 ± 0.8 ‰, n = 32) [43]. The majority of animals (except dogs) from the same site demonstrate consistency with a C_3_ diet and relatively low nitrogen isotope values (Fig 3A and 3B; mean *δ*^13^C = -19.0 ± 2.4 ‰; *δ*^15^N = 5 ± 1.4 ‰, n = 50). Carbon isotope ratios in humans seemingly suggest that humans directly consumed millet as a staple food, perhaps on a daily basis. Nitrogen isotope ratios on the other hand, suggest that the animal protein consumption at Xinglonggou was also significant, with a human-animal collagen offset of about 5 ‰ in *δ*^15^N values.

To further explore these localized dietary patterns observed in bulk collagen data, we used an isotope mixing model to quantify the importance of C_4_ plants to human diets at this site. The results suggest that the proportional contribution of C_4_ plants (likely millet) to human diet at Xinglonggou was between 52–62% (95% CI; Fig 3C, S5 Table). Herbivores formed the second most important human dietary item, accounting for approximately 33–46%. These results confirm that humans in the Xinglonggou community relied on C_4_ plants as a staple food. Nonetheless, when dietary reconstruction is based on bulk collagen isotopic determinations, informative variation at the molecular level is masked. Future research at the compound specific level that separates essential and non-essential amino acid isotope values could be undertaken to confirm or refute the validity of these interpretations derived from bulk collagen isotope data.

#### 5000–2000 cal BC

**Fig 4:**
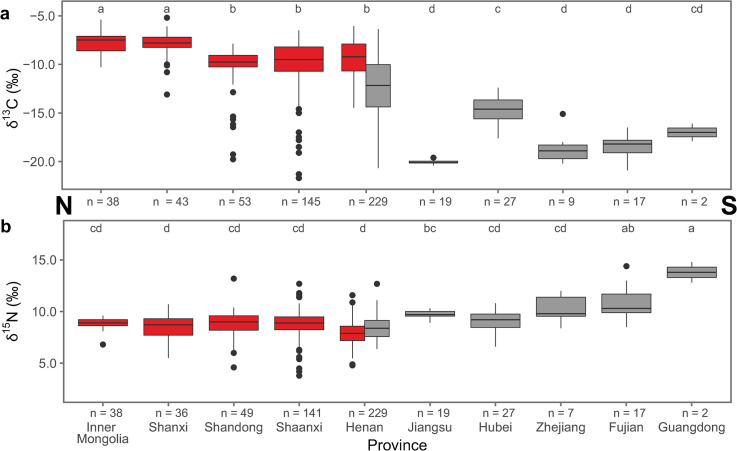
Isotope data by province, 5000–2000 cal BC. Boxplots of human bone collagen *δ*^13^C (a) and *δ*^15^N values (b) by province from sites that date to between 5000–2000 cal BC arranged from north (left) to south (right). Regions are differentiated by color: Loess Plateau (red) and Yangtze-Huai (gray). Provinces sharing a letter are not significantly different (Tukey’s HSD). Statistics are summarized in S3A and S3B Table.

Between 5000 and 2000 cal BC, isotope data from the Loess Plateau and the Yangtze-Huai Region reveal a more pronounced north-south distinction in human diets. Limited data are available from the Continental Interior. Humans from the Yangtze-Huai Region preserve isotopic signatures consistent with C_3_-dominated diets, while humans from the Loess Plateau present isotopic values suggesting they consumed a varying degree of C_4_ plant foods (Fig 2D–2F). There is a statistically significant difference in the δ^13^C and δ^15^N values from these two regions (Welch’s ANOVA, δ^13^C: F_1,586_ = 291.1, *p* < 2.2e-16; δ^15^N: F_1,569_ = 20.9, *p* = 5.785e-06). Twenty-seven out of thirty populations from the Loess Plateau show significant consumption of C_4_ plants (δ^13^C > -12‰, see Table 3). High δ^13^C values can also be caused by significant consumption of marine resources, making it difficult to distinguish between C_4_ and marine dietary inputs for coastal sites as in Shandong and the Lower Yangtze, where marine resources were abundant in the archaeological record [44]. The three Loess Plateau sites that do not exhibit dominant C_4_ consumption at this time are all located in more southerly provinces that border on the Yangtze-Huai Region (Shandong and Shaanxi), suggesting that there is probably northward expansion of rice cultivation at this time (Fig 4A). In the Yangtze-Huai Region, seven out of thirteen populations have isotope values consistent with a predominantly-C_3_ diet (δ^13^C < -17‰, see Table 3). The other six populations are consistent with a predominantly-C_4_ diet. The latter group comes from the southern Henan and Hubei provinces, and likely reflects the southern expansion of millet cultivation in this period. Several individuals with extremely high δ^15^N values in the Yangtze-Huai region (Fig 2F) come from coastal sites at which marine resources were likely being consumed [45, 46].

**Fig 5:**
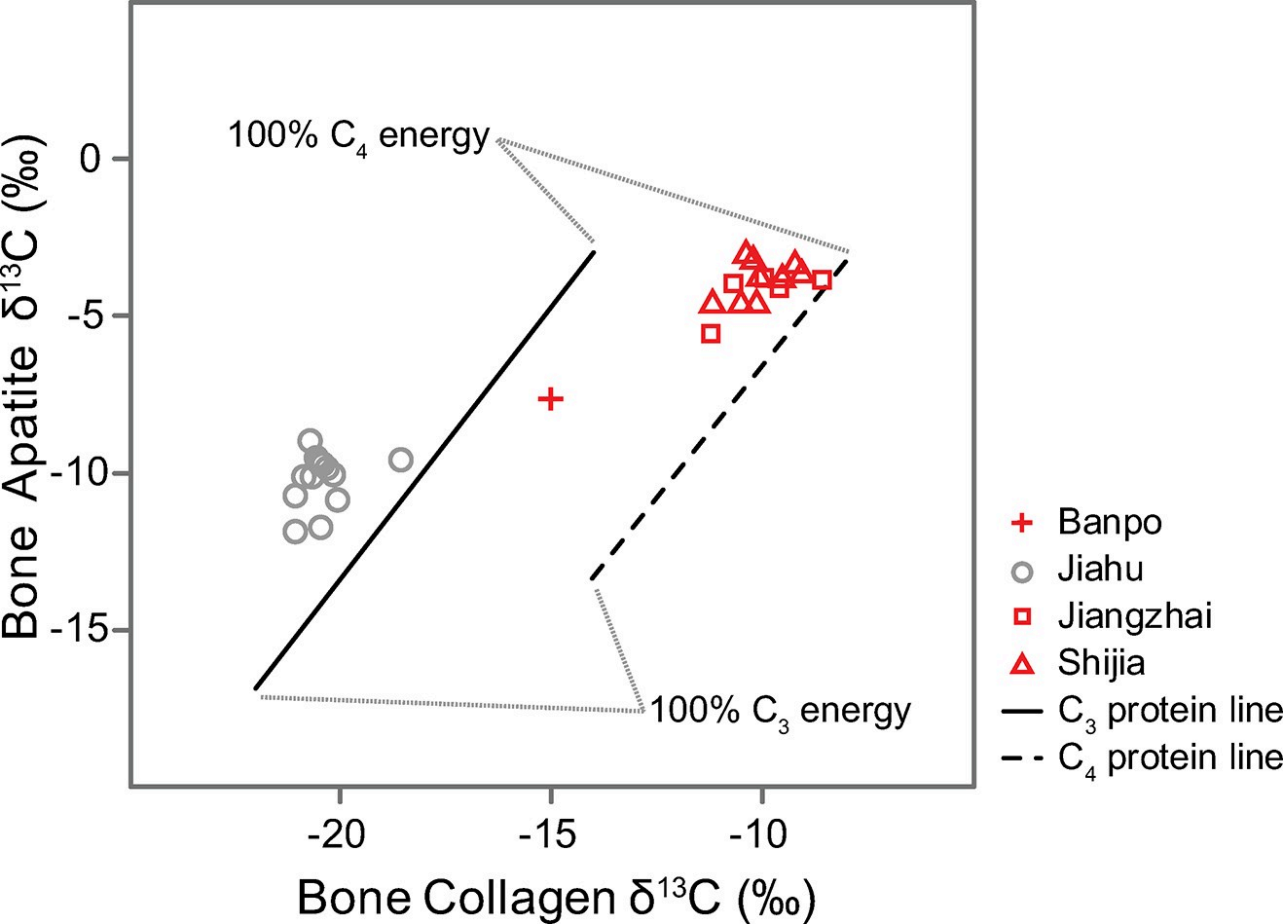
Kellner and Schoeninger’s approach. *δ*^13^C_apatite_ and *δ*^13^C_collagen_ measured in archaeological humans from one site dating to >5000 cal BC in the Yangtze-Huai region (gray, Jiahu) and three sites from the Loess Plateau dating to between 5000 and 2000 cal BC (red). Humans plotting along the C_3_ regression line are interpreted to consume primarily C_3_ protein, while those along the C_4_ regression line consume C_4_ protein. The position on the line along the y-axis is further indicative of the energy source, with low apatite *δ*^13^C values corresponding with C_3_ energy sources and high apatite *δ*^13^C values corresponding with C_4_ energy sources.

Some interesting patterns emerged in the collagen versus apatite δ^13^C plot, using Kellner and Schoeninger’s approach as described in the methods (Fig 5). At Jiahu, humans plot along the C_3_ protein line, but their position on the y-axis (~ -10‰) suggests that their energy comes from a mixture of C_3_ and C_4_ resources. Humans from Jiangzhai and Shijia, on the other hand, plot more closely to the C_4_ protein line and their position along the y-axis, with apatite δ^13^C values > -5‰, suggests their dietary energy is derived primarily from C_4_ energy sources. Both of these sites are located on the Loess Plateau and these results help to clarify that some humans from this time period and region were likely consuming fully C_4_ diets. The individual from Banpo, another Loess Plateau site, tells a slightly different story because they fall between the C_3_ and C_4_ protein lines, suggesting a mixed protein diet; their apatite δ^13^C value is similarly suggestive of mixed C_3_ and C_4_ energy sources. Although this method is not quantitative, it nonetheless allows for energy and protein resources to be evaluated separately, allowing for a deeper understanding of past human diet than bulk collagen isotope values provide. Indeed, these data suggest that later in the period of 5000–2000 cal BC, some humans on the Loess Plateau were consuming millet directly as well as animals provisioned with millet (Jiangzhai and Shijia).

#### 2000 cal BC– 220 cal AD

**Fig 6:**
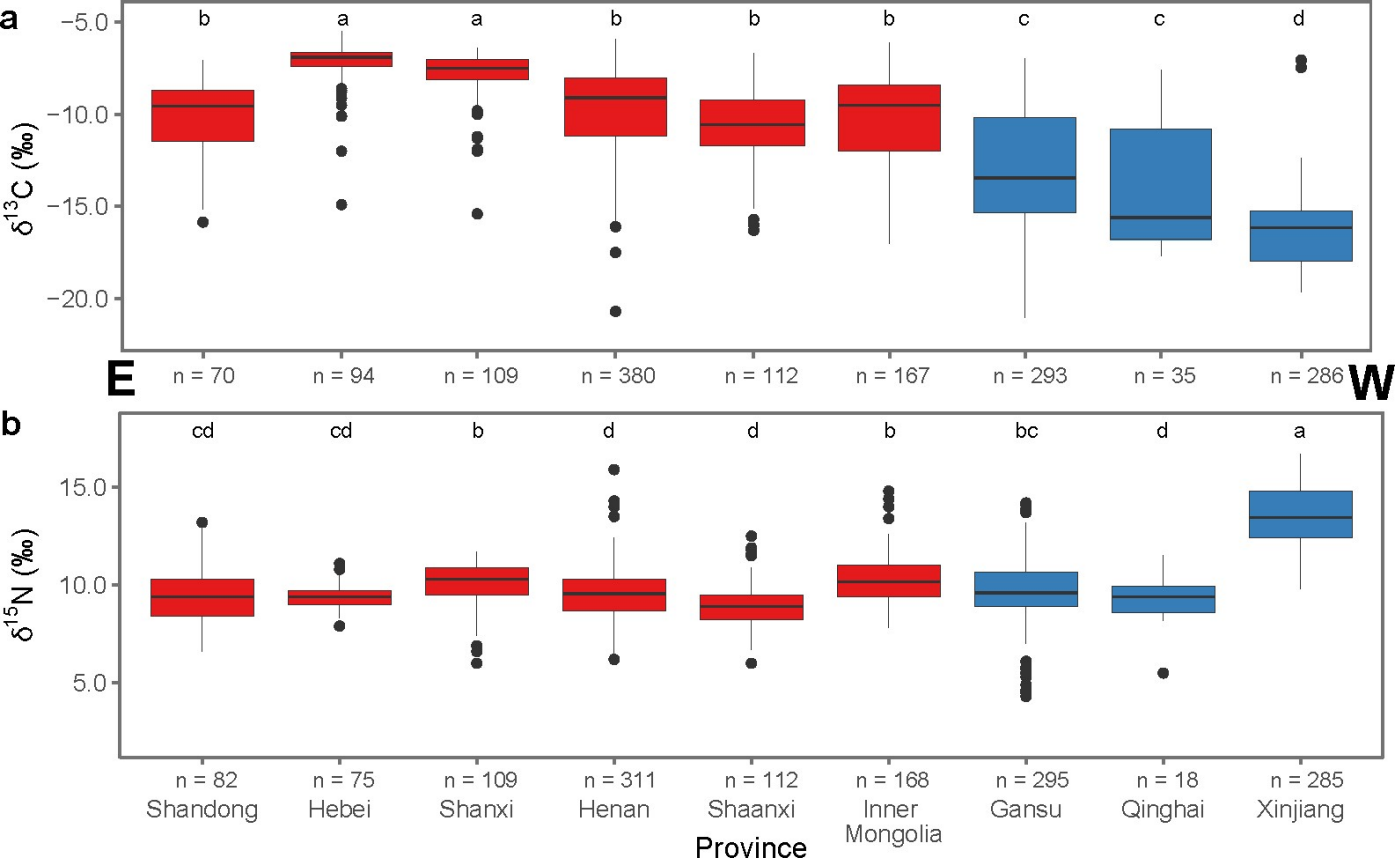
Isotope data by province, post-2000 cal BC. Boxplots of human bone collagen *δ*^13^C (a) and *δ*^15^N values (b) by province from sites that date to post-2000 cal BC arranged from east (left) to west (right). Regions are differentiated by color: Loess Plateau (red) and Continental Interior (blue). Provinces sharing a letter do not have significantly different means (Tukey’s HSD). Statistics are summarized in S3C and S3D Table.

Between 2000 cal BC and 220 cal AD, China’s staple food system experienced a major shift resulting from the introduction of wheat and barley (both are C_3_ plants) [47–49]. The compiled isotopic data reflect distinct dietary choices between the prehistoric communities in the Loess Plateau and the Continental Interior (Fig 2G–2I). In the Loess Plateau, other than a few exceptional individuals from Henan Province, humans show isotopic signatures consistent with predominantly-C_4_ or mixed C_3_-C_4_ consumption. Conversely, humans from the Continental Interior exhibit a broader spectrum of dietary habits including predominantly-C_3_, mixed C_3_-C_4_, and predominantly-C_4_ diets. The two regions show statistically significant differences in *δ*^13^C and *δ*^15^N values (*δ*^13^C: Welch’s ANOVA, F_1,1548_ = 1113.8, *p* < 2.2e-16; *δ*^15^N: Welch’s ANOVA, F_1,1454_ = 335.13, *p* < 2.2e-16). Human data from 39 out of 43 sites from the Loess Plateau suggest that millet consumption was very significant (*δ*^13^C > -12‰), while human data from 19 out of 28 sites from the Continental Interior are consistent with C_3_ or C_3_-C_4_ mixed diets (*δ*^13^C < -12‰). The significantly different *δ*^15^N values between the two regions could be the result of a combination of several factors, including variable animal protein input, differences in crop δ^15^N values caused by variable soil ^15^N enrichment, or aridity in the Continental Interior. The earlier north to south divide in staple crop use is accompanied by a new divide between the east and the west (see Fig 6).

### Gendered consumption

**Fig 7:**
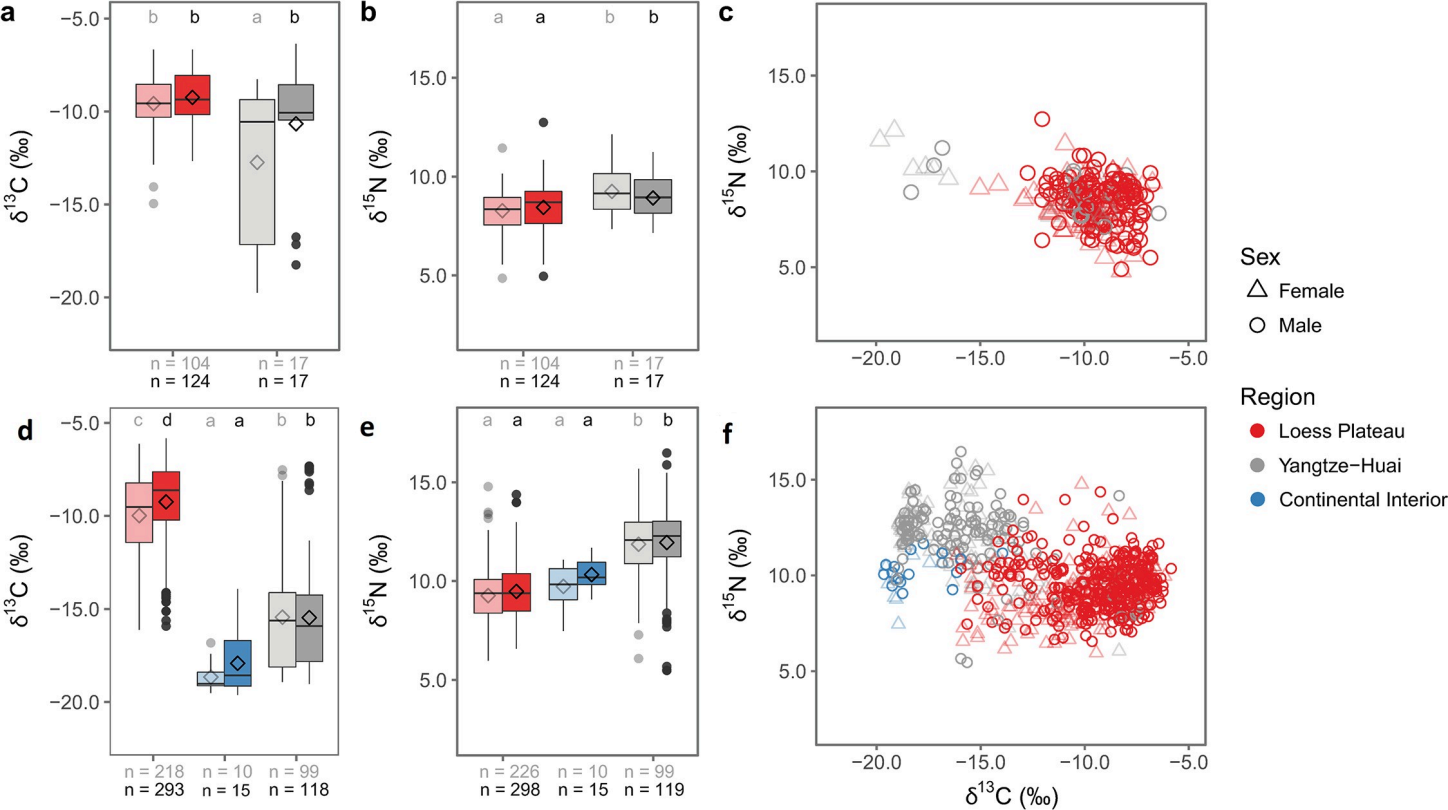
Isotope data by sex. Boxplots and scatterplots of human bone collagen *δ*^13^C and *δ*^15^N values by sex from sites occupied between 5000–2000 cal BC (a-c), and post-2000 cal BC (d-f). Regions are differentiated by color and sex by shape and shade (female = lighter shaded, open triangles, male = darker shaded, open circles). Boxplot components are described in Fig 2. N-values listed at the base of the boxplots are divided by female (gray) and male (black). In panels a, b, d, and e, groups sharing a letter do not have significantly different means (Tukey’s HSD). Statistics are summarized in S4A and S4B Table.

We next consider the differences in staple consumption between males and females between 5000 and 2000 cal BC. In the Loess Plateau, females and males do not have significantly different *δ*^13^C and *δ*^15^N values (Fig 7A and 7B). In the Yangtze-Huai Region, no significant difference in *δ*^15^N is observed, but significant differences are observed in *δ*^13^C values (*p* = 0.0014, Fig 7A and 7B, S4A and S4B Table), with males exhibiting higher carbon isotope values. This difference is primarily driven by regional variations within the Yangtze-Huai Region; when sexed individuals are compared within provinces, no significant differences are observed (Fig A in S1 File, S4C and S4D Table). It does not appear that social customs prohibited the consumption of C_4_-plants or animals consuming C_4_ products by females in either the Loess Plateau or Yangtze-Huai Region during 5000–2000 cal BC, despite the fact that males consumed these foods to a higher degree than females in both regions.

After 2000 cal BC, males exhibited higher *δ*^13^C and *δ*^15^N values than females in all three regions (Fig 7D and 7E), although the Loess Plateau is the only region where male and female *δ*^13^C values differ significantly (*p* = 0.01, S4E and S4F Table). Because there is a risk of conflating gender differences with differences in social status, particularly when sample sizes are small, we focus our discussion on the Loess Plateau, where sample sizes are greatest. Lower *δ*^13^C values in females from the Loess Plateau (n = 218) could indicate that females had greater access to newly introduced C_3_ crops than males (n = 293). When gendered differences are considered at the provincial level, differences (although not significant) are evident in several provinces where males display higher access to C_4_ resources and protein products (e.g., Shandong, Henan, Shaanxi, Inner Mongolia and Gansu; Fig B in S1 File, S4G and S4H Table). Only in Henan do males have significantly higher *δ*^13^C values than females, which has been clearly documented at sites in the region [50].

## Discussion

Our results suggest that both environmental and cultural-culinary conditions contributed significantly to the formation of staple cuisines in China. We shall next consider the observed isotopic patterns in two temporal and spatial dimensions. Before 2000 cal BC, the north-and-south dietary division will be considered in the context of regional variations of subsistence activities, which are partly driven by differences in environmental conditions. After 2000 cal BC, the introduction of crops originating from southwestern Asia resulted in an additional east-to-west gradient in the degree of incorporation of wheat and barley in human diets. We shall explore this pattern in relation to culinary traditions and emphasize the incompatibility of novel exotic grains with local culinary practice.

It is no exaggeration to say the millennium between 6000 and 5000 cal BC is crucial to understanding the origins of farming activities in East Asia [14, 16]. The north and south dietary divergence observed in this period is better understood as a difference in the spectrum of subsistence activities, rather than as separated peoples. In the Yangtze-Huai Region, human carbon isotope values are consistent with a C_3_-plant dominated diet, which likely consisted of C_3_ resources identified in the archaeobotanical record (i.e. rice, fruits, tubers, nuts) [24]. In the Loess Plateau, however, humans relied on C_4_ foods. Broomcorn and/or foxtail millet were documented at all four northern sites (or associated cultural sites) in high quantities [13, 51]. No other C_4_ cereal has been identified in the plant macrofossil assemblages from this time period. There is microbotanical evidence for job’s tears (a C_4_ plant) at Xinglonggou [52], however, given the tropical adaptation of genus *Coix*, job’s tears were unlikely to be cultivated on a large enough scale to become a staple cereal 7500 years ago. The tradition of consumption of C_4_ crops as staple foods emerged in this period and was particularly pronounced among the Xinglongwa cultural communities. At Xinglonggou, we estimated the proportional contribution of C_4_ plants to human diet to be greater than 50%, nearly two-times more significant than herbivores, the second most important dietary resource.

Above all, the distinct subsistence modes between north and south in Neolithic China are driven by regional environmental differences. The lower catchment of the Yangtze and Huai Rivers was an intricate deltaic wetland crisscrossed by hundreds of distributaries, merging and diverging with seasonal flooding. People in this region relied overwhelmingly on wetland resources, including rice–an aquatic plant—for their subsistence. In contrast, landscapes in the north form a single relatively uniform semi-arid zone across the Loess Plateau. From early on, millet cultivation became the key component of agrarian based subsistence in the Loess Plateau. Within this perspective, the broad spectrum of subsistence activities in the Yangtze-Huai within an environmental mosaic consisting of swamps, marshes, fens and wetlands can be seen as the mirror image of unified agrarian practices based on millet grain in the northern Loess Plateau. That is, both of these highly sustainable systems in the north and south took advantage of the subsistence options their landscape setting provided. And this arrangement seems to have persisted for another 3000 years (5000–2000 cal BC). The regional difference in dietary tradition between north and south, along with the variation within each region, challenges the conventional “stages” of shifting modes of subsistence–hunting, foraging, pastoralism, and farming–in an evolutionary framework. Both historical and archaeological evidence shows that peoples moved fairly readily between distinctive modes of subsistence and the same people might have practiced more than one subsistence mode in a single lifetime [53, 54]. In China as elsewhere, it seems early peoples combined subsistence modes in a number of innovative hybrids that co-existed over thousands of years. The north-south dietary distinction in China resonates with the conceptual distinction in southwest Asia between the northern “Hilly Flanks” and the southern Mesopotamian alluvium highlighted by James Scott [54].

The rapid adoption of wheat and barley as staple foods in the Continental Interior by 2000 cal BC contrasts the very gradual pace of the adoption of these western crops in the Loess Plateau. In a recent review focused on northern China, the authors noted that the shift from a C_4_-dominated diet to a mixed C_3_-C_4_ diet at this time was concurrent with “Holocene Event 3” at 4200 BP (2,250 BC) [55]. A global aridification event may well be part of the explanation of the readiness of communities in the Continental Interior to accept wheat and barley as new staples. Nonetheless, the question remains—what delayed the adoption of wheat and barley in the Loess Plateau? As discussed elsewhere, one plausible social explanation is that in the early stages of their adoption in the Loess Plateau, these crops were exclusively used by the few—such as elites, ritual specialists or others—rather than the many [56]. But this is not the only explanation.

An alternative interpretation lies in the deep-seated East and West culinary distinction. As established, boiling and steaming of grains and other foods appear to have been and remained the predominant East Asian methods for preparing foods. By contrast, cereals in southwestern and Central Asia such as wheat and barley were processed for a flour-focused food system. Such an East-West culinary distinction can be traced back to the pre-agricultural Palaeolithic [57]. These culinary preferences had consequences for the selection of grain quality and features, with gluten protein being the target of selection in west Eurasia for making bread, and starch properties being selected in East Asia for the function of boiling-steaming. It has been hypothesized that the western boundary of the boiling-steaming culinary tradition appears to correspond approximately to the geographic range of the summer monsoon [57]. In other words, the people of the Loess Plateau and Continental Interior each belonged to two distinct culinary systems: the boiling-and-steaming cultures in the East and grinding-and-baking cultures in the West. The gradual adoption of western grains (wheat and barley) and the isotopic evidence associated with it could be understood in this context. The dispersal of crops into new areas was not necessarily accompanied by the spread of the culinary traditions from their regions of origin. Novel grains could instead be incorporated into existing local practices of food processing. In the case of wheat, it has been illustrated this incorporation may have exerted selection on the crops for phenotypic traits adapted to the eastern cooking traditions [56]. In southeast Asia, the preference for cultivation of cereals that show within-species variation for stickiness of the cooked grain are typified by the eastern boiling-and-steaming cultures [58]. In both cases, it is plausible that the novel grains from the West (i.e. wheat and barley) might be initially “rejected” as a staple grain because of their incompatibility with local culinary practice, and this is consistent with the isotopic results showing a significant delay in human consumption of wheat and barley as a staple food in the Loess Plateau. Within the context of symbolism and social use of food [1], culinary traditions are often related to kinship and family structure, and that was the case in southeast Asia with the sticky food culture [58]. In the post-2000 BC Loess Plateau, newly introduced staple cereals from the West were consumed by females to a greater degree than males. This hints at the gender roles in the context of social status of grains and food processing with the female sector of the society being the primary agent of the process, pioneering innovations in culinary practice.

## Conclusion

Modern Chinese cuisine formed over thousands of years through the development of diverse regional subsistence systems and cuisines, which were further influenced by food traditions from other parts of the world. Our results help to illustrate the ways in which both environment and culture contributed to shaping the Chinese staple food system over the past 8000 years. A distinct north versus south divide in Chinese ancient staple cuisines was already evident isotopically between 6000–5000 cal BC and became more pronounced between 5000–2000 cal BC. We infer that this pattern is better understood as a difference in the spectrum of subsistence activities, which was partly driven by environmental differences between the Loess Plateau and the Yangtze-Huai region. The introduction of wheat and barley from southwestern Asia after 2000 cal BC resulted in the development of an additional east to west gradient in the degree of incorporation of the different staple products into human diets. We argue the regional differences in dietary tradition between and within the three broad regions throughout the Neolithic and the Bronze Age could not be understood in the conventional “stages” of shifting modes of subsistence: hunting-foraging-pastoralism-farming. Instead the same people might have practiced more than one subsistence mode and combined them in a number of innovative hybrids that co-existed over thousands of years. The rapid adoption of wheat and barley as staple foods in the Continental Interior by 2000 cal BC contrasts the very gradual pace of the adoption of these western crops in the Loess Plateau. Apart from the possible environmental and social drivers, we explored a third explanation that these novel grains may have at first been ignored as a staple grain because of their incompatibility with local culinary practice; people of the Loess Plateau belonged to a boiling-and-steaming culture while those in the Continental Interior belonged to a grinding-and-baking culture into which wheat and barley were more readily folded. Finally, in some cases in the Loess Plateau, newly introduced staple cereals from the West were consumed by females to a greater extent than males, suggesting that the female sector of society may have pioneered the innovations in culinary practice.

## Supporting information

S1 File Supplementary material: Gender differences at the provincial level. (DOCX)

S2 File R scripts used in this study. (DOCX)

S1 Table Published carbon and nitrogen isotope data used in this study. Data are from archaeological human skeletal remains (n = 2448) from 128 sites across China.
(XLSX)

S2 Table Summary of key archaeobotanical studies from selected sites cross China. See [2] for detailed site/assemblage information.
(XLSX)

S3 Table **A-D**. ANOVA results by province. Results of comparisons of human *δ*^13^C (Table A in S3 Table) values and *δ*^15^N (Table B in S3 Table) by province in time period II (5000–2000 cal BC), and results of comparisons of human *δ*^13^C (Table C in S3 Table) values and *δ*^15^N (Table D in S3 Table) by province in time period III (2000 cal BC– 220 cal AD).
(XLSX)

S4 Table **A-H**. ANOVA results by sex. Results of comparisons of male and female δ^13^C (Table A in S4 Table) and *δ*^15^N (Table B in S4 Table) values by region in time period II (5000–2000 cal BC); ANOVA comparisons of human δ^13^C (Table C in S4 Table) and *δ*^15^N (Table D in S4 Table) values by sex and province in time period II (5000–2000 cal BC); ANOVA comparisons of male and female δ^13^C (Table E in S4 Table) and *δ*^15^N (Table F in S4 Table) values by region in time period III (2000 cal BC– 220 cal AD); and ANOVA comparisons of human δ^13^C (Table G in S4 Table) and *δ*^15^N (Table H in S4 Table) values by sex and province in time period III (2000 cal BC– 220 cal AD).
(XLSX)

S5 Table Mixing model results for Xinglonggou humans. Results from the model run highlighted in green are reported in the text.
(XLSX)
