# Supplementary material for: The prehistoric roots of Chinese cuisines: Mapping staple food systems of China, 6000 BC–220 AD
Source: PLoS One. 2020 Nov 4;15(11):e0240930. doi: 10.1371/journal.pone.0240930 (PMC7641357; doi:10.1371/journal.pone.0240930)
Supplement: S1 File — (DOCX) [file pone.0240930.s001.docx]

**S1 File. Supplementary Material**

***Gender differences at the provincial level***

When considering sexed individuals from sites dating to between 5000 and 2000 cal BC (Figure 4 a-c), there is no significant difference observed in $\delta$^13^C and $\delta$^15^N between females and males in the Loess Plateau. In the Yangtze-Huai Region, no significant difference in $\delta$^15^N is observed, but significant differences are observed in $\delta$^13^C values (*p* = 0.0014, Table S10-11), with males exhibiting higher carbon isotope values. When regional gendered difference is considered at the provincial level (see Figure S3 and Table S12-13), differences in $\delta$^13^C values are observed in Henan, with males presenting higher carbon isotope values. Difference in $\delta$^15^N is also observed in a number of provinces, with males exhibiting higher nitrogen isotope values in Shaanxi and northern Henan and females exhibiting higher nitrogen isotope values in Shanxi, Shandong and Fujian. However, none of these differences are significant.

During the time between 2000 cal BC and 220 cal AD, males exhibited higher $\delta$^13^C and $\delta$^15^N values than females in all three regions (Figure 8 4-f), though the Loess Plateau is the only region where differences in male and female $\delta$^13^C values are significant (*p* = 0.01, Table S14-15). When regional gendered difference is considered at the provincial level (see Figure S4 and Table S16-17), differences (though not significant) are evident in several provinces (e.g., Henan, Shaanxi, Inner Mongolia and Gansu) where males display higher nitrogen isotope values and, at some sites in Shandong, Shanxi, Henan, Shaanxi, Inner Mongolia and Gansu higher carbon isotope values. Only in Henan do males have significantly higher $\delta$^13^C values than females.


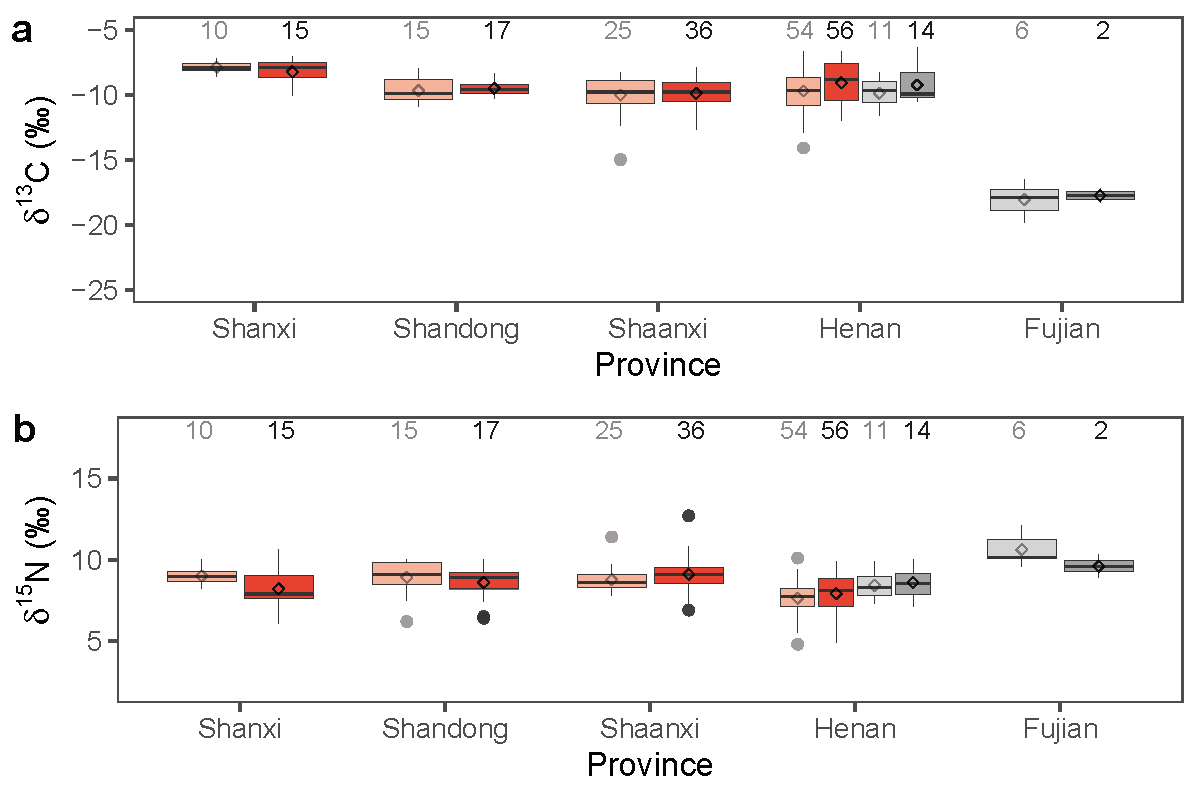


**S1 File Fig A. Sex differences within provinces (5000-2000 cal BC).** Boxplots of human bone collagen $\delta$^13^C (a) and $\delta$^15^N values (b) by sex and province from sites dating to 5000-2000 cal BC arranged from north (left) to south (right). Regions are differentiated by color: Loess Plateau (red) and continental interior (blue); and sex by shade female (lighter shaded) male (darker shaded). Boxplots illustrate minimum, first quartile, median, third quartile, and maximum; means are depicted as hollow black diamonds and outliers as black dots.

*
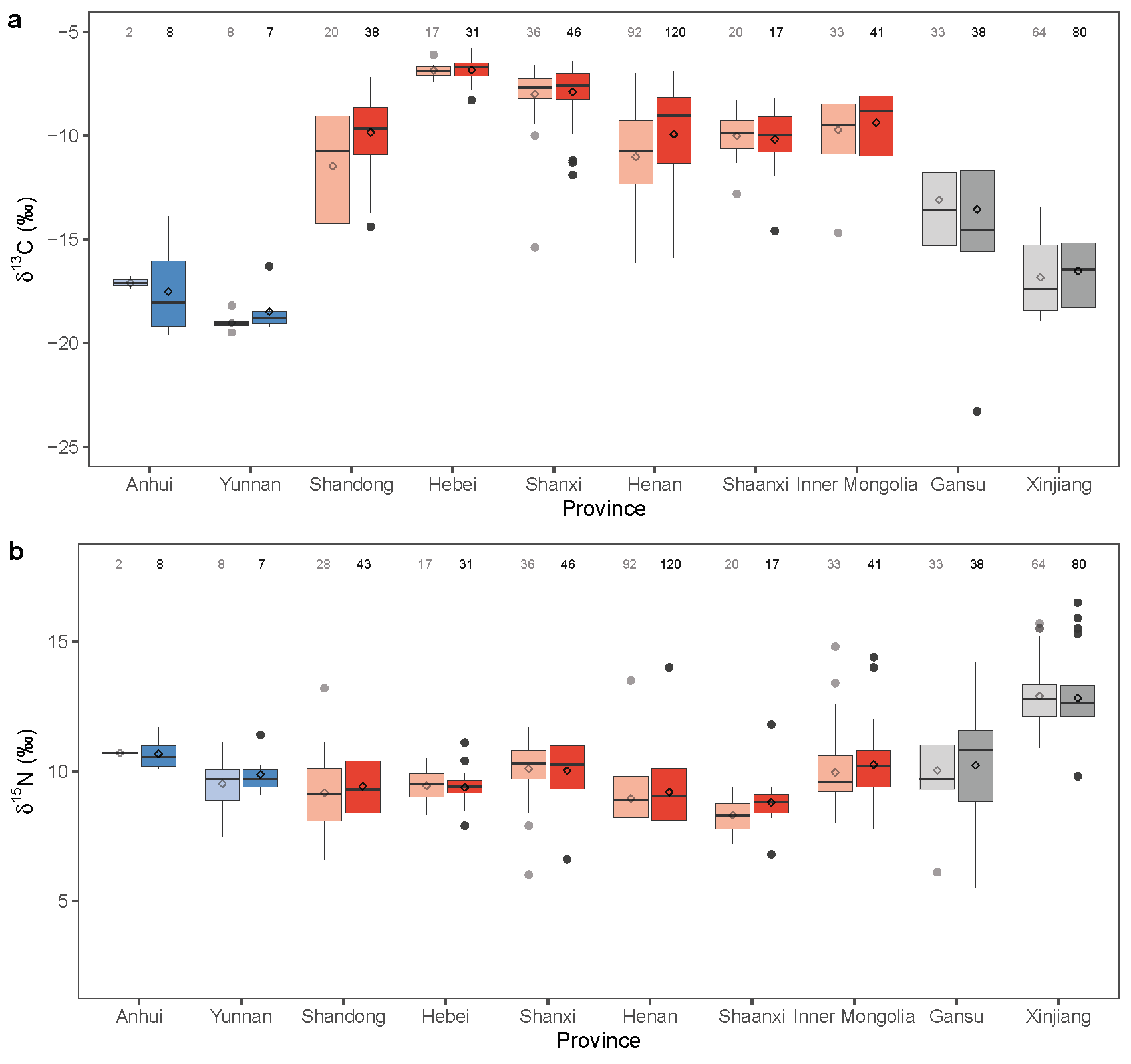
*

**S1 File Fig B. Sex differences within provinces (post 2000 cal BC).** Boxplots of human bone collagen $\delta$^13^C (a) and $\delta$^15^N values (b) by sex and province from sites dating to post 2000 cal BC arranged from east (left) to west (right). Regions are differentiated by color: Loess Plateau (red), continental interior (blue), and Yangze-Huai (gray); and sex by shade: female (lighter shaded) male (darker shaded). Boxplots illustrate minimum, first quartile, median, third quartile, and maximum; means are depicted as hollow black diamonds and outliers as black dots.
