# Supplementary material for: The prehistoric roots of Chinese cuisines: Mapping staple food systems of China, 6000 BC–220 AD
Source: PLoS One. 2020 Nov 4;15(11):e0240930. doi: 10.1371/journal.pone.0240930 (PMC7641357; doi:10.1371/journal.pone.0240930)
Supplement: S2 File — (DOCX) [file pone.0240930.s002.docx]

### Mapping Staple Cuisines

### Rachel E. B. Reid / Virginia Tech / rebreidATvtDOTcom

### 2020

### This code accompanies the article entitled "The prehistoric roots of Chinese cuisines:

### Mapping staple food systems of China, 6000 BC - 220 AD" by Xinyi Liu and Rachel Reid

################

### CONTENTS ###

################

### Libraries, functions, and definitions ###

### Data Summary ###

### Pre-5k ###

# Pre-5k plots by region

# Parametric Stats; pre5k

### Group II ###

# Parametric Stats; group II regions

# Group II plots by region

# Group II boxplots ordered N to S

# Parametric Stats; group II provinces

### Group III ###

# Parametric Stats; group III regions

# Group III plots by region

# Group III boxplots ordered E to W

# Parametric stats; group III provinces

### Sex Differences ###

# Group II Sex Differences

# Parametric stats; Group II sex and region

# Group III Sex Differences

# Parametric stats; Group III sex and region

# Sex differences by Province - Group II

# Sex Differences by Province - Group III

#############################################

### Libraries, functions, and definitions ###

#############################################

library(tidyverse)

library(ggthemes)

library(extrafont)

library(gridExtra)

library(cowplot)

library(agricolae)

library(car)

library(multcompView)

library(lsmeans)

#Write function to give N

give.nC <- function(x){

return(c(y = -5.0, label = length(x)))

}

give.nN <- function(x){

return(c(y = 18, label = length(x)))

}

########################

##### Data Summary #####

########################

# Set your working directory to the proper source file location on

# on your computer: setwd("C:/Users/rbrow/Review paper/Data/Analyses")

# The data file referenced here is a .csv version of S1 Table,

# included with the paper. Note column names used below.

all<-read.csv(file="AllData2019.csv",sep=",",header=TRUE, stringsAsFactors = FALSE)

summary(all)

str(all)

all<-rename(all, Site = Ô..Site)

all$Site <- as.factor(all$Site)

all$Province <- as.factor(all$Province)

all$Region <- as.factor(all$Region)

all$Time_Group <- as.factor(all$Time_Group)

all$d13C <- as.numeric(all$d13C)

all$d15N <- as.numeric(all$d15N)

all$CN <- as.numeric(all$CN)

all$Reference <- as.factor(all$Reference)

all$Sex <- as.factor(all$Sex)

# filter out data with poor CN ratio

all$CN[is.na(all$CN)] <- 0

all <- filter(all, CN < 3.7)

## Plot all the data together

biplot <- ggplot(all,aes(d13C,d15N)) +

geom_point(aes(color = Region, shape = Time_Group), size=2) +

scale_color_manual(values = c("#E41A1C", "#999999", "#377eb8")) +

scale_shape_manual(values=c(21,22,23)) +

ylim(0,18)+xlim(-30,0) + #set the plot area

labs(x=expression(paste(delta^{13},"C", " \u0028","\u2030","\u0029"))) + #label the x axis

labs(y=expression(paste(delta^{15},"N"," \u0028","\u2030","\u0029"))) + #label the y-axis

labs(title="All China")

biplot

## Make tables by time period

pre5k <- filter(all, Time_Group == "I")

groupII <- filter(all, Time_Group == "II")

groupIII <- filter(all, Time_Group == "III")

pre5k_Site <- pre5k %>%

filter(!is.na(d15N)) %>%

group_by(Site, Reference) %>%

summarise(

Meand15N = mean(d15N),

SDd15N = sd(d15N),

Meand13C = mean(d13C),

SDd13C = sd(d13C),

n = n()

)

#write.csv(pre5k_Site, file = "pre5k_Site.csv")

groupII_Site <- groupII %>%

group_by(Site, Region) %>%

summarise(

Meand15N = mean(d15N, na.rm = TRUE),

SDd15N = sd(d15N, na.rm = TRUE),

Meand13C = mean(d13C, na.rm = TRUE),

SDd13C = sd(d13C, na.rm = TRUE),

n = n()

)

#write.csv(groupII_Site, file = "groupII_Site.csv")

groupIII_Site <- groupIII %>%

group_by(Site, Region) %>%

summarise(

Meand15N = mean(d15N, na.rm = TRUE),

SDd15N = sd(d15N, na.rm = TRUE),

Meand13C = mean(d13C, na.rm = TRUE),

SDd13C = sd(d13C, na.rm = TRUE),

n = n()

)

#write.csv(groupIII_Site, file = "groupIII_Site.csv")

#################

##### pre5k #####

#################

#### Pre-5k plots by region ####

pre5k <- filter(all, Time_Group == "I")

pre5k <- filter(pre5k, Site != "Dingshishan") # Remove samples without d15N values

pre5k$Site <- as.character(pre5k$Site)

pre5k$Site <- factor(pre5k$Site, levels=unique(pre5k$Site))

C_bp_pre5<-ggplot(pre5k, aes(Region, d13C)) + geom_boxplot(aes(fill = factor(Region))) +

stat_summary(fun.y=mean, geom="point", shape=5, size=3) +

scale_fill_manual(values = c("#E41A1C","#999999")) +

theme_few(base_size=12) +

scale_y_continuous(breaks = c(0, -5, -10, -15, -20, -25, -30),

labels = c("0","-5.0", "-10.0","-15.0","-20.0","-25.0","-30.0"),

limits = c(-25,-5)) +

labs(y=expression(paste(delta^{13},"C"," \u0028","\u2030","\u0029"))) + #label the y-axis

theme(legend.position="none") +

stat_summary(fun.data = give.nC, geom = "text", size = 3)

C_bp_pre5

N_bp_pre5<-ggplot(pre5k, aes(Region, d15N)) + geom_boxplot(aes(fill = factor(Region))) +

stat_summary(fun.y=mean, geom="point", shape=5, size=3) +

scale_fill_manual(values = c("#E41A1C","#999999")) +

theme_few(base_size=12) +

scale_y_continuous(breaks = c(0, 5, 10, 15, 20),

labels = c("0","5.0", "10.0","15.0","20.0"),

limits = c(2,18)) +

labs(y=expression(paste(delta^{15},"N"," \u0028","\u2030","\u0029"))) + #label the y-axis

theme(legend.position="none") +

stat_summary(fun.data = give.nN, geom = "text", size = 3)

N_bp_pre5

# Plot just the pre-5k sites

pre5k_plot <- ggplot(pre5k,aes(d13C,d15N)) +

geom_point(aes(color = Region, shape = Province), size=2, stroke=0.5) +

scale_shape_manual(values=c(2,3,5,6)) +

scale_color_manual(values = c("#E41A1C","#999999"), labels = c("Loess Plateau", "Yangtze-Huai")) +

scale_y_continuous(breaks = c(0, 5, 10, 15, 20),

labels = c("0","5.0", "10.0","15.0","20.0"),

limits = c(2,18)) +

scale_x_continuous(breaks = c(0, -5, -10, -15, -20, -25),

labels = c("0","-5.0", "-10.0","-15.0","-20.0","-25.0"),

limits = c(-22,-5)) +

labs(x=expression(paste(delta^{13},"C", " \u0028","\u2030","\u0029"))) + #label the x axis

labs(y=expression(paste(delta^{15},"N"," \u0028","\u2030","\u0029"))) + #label the y-axis

theme_few(base_size=12)

pre5k_plot

pre5k_plots <- plot_grid(C_bp_pre5,N_bp_pre5,pre5k_plot,

ncol = 3,

rel_widths = c(1/4,1/4,1/2),

labels = "auto")

pre5k_plots

#ggsave(filename="pre5k_plots.pdf", width = 10, height = 3, plot=pre5k_plots)

#### Parametric Stats; pre5k ####

# Are regions different?

fit_C <- aov(d13C ~ Region, data = pre5k)

fit_C

summary(fit_C)

hist(fit_C$residuals)

fit_N <- aov(d15N ~ Region, data = pre5k)

fit_N

summary(fit_N)

hist(fit_N$residuals)

####################

##### Group II #####

####################

groupII <- filter(all, Time_Group == "II")

groupII <- filter(groupII, Region != "C")

str(groupII)

# Plot by N vs. S

NvsS<-ggplot(groupII,aes(d13C,d15N)) +

geom_point(aes(color=Region), size=3, shape=21, stroke = 1.5) +

scale_color_brewer(palette="Set1", labels = c("Loess Plateau", "Yangtze")) +

ylim(2,16)+xlim(-23,-4) + #set the plot area

labs(x=expression(paste(delta^{13},"C", " \u0028","\u2030","\u0029"))) + #label the x axis

labs(y=expression(paste(delta^{15},"N"," \u0028","\u2030","\u0029"))) + #label the y-axis

labs(title="Loess Plateau vs. Yangtze, 5000-2000 BC") +

theme_few(base_size=12)

NvsS

#### Parametric Stats; group II regions ####

# Are the regions significantly different?

fit_C <- aov(d13C ~ Region, data = groupII)

fit_C

summary(fit_C)

hist(fit_C$residuals)

leveneTest(d13C ~ Region, data = groupII)

oneway.test(d13C ~ Region, data = groupII, var.equal = FALSE)

model <- lm(d13C ~ Region, data = groupII)

oneway_C <- Anova(model, Type ="II", white.adjust=TRUE)

oneway_C

fit_N <- aov(d15N ~ Region, data = groupII)

fit_N

summary(fit_N)

hist(fit_N$residuals)

leveneTest(d15N ~ Region, data = groupII)

oneway.test(d15N ~ Region, data = groupII, var.equal = FALSE)

model <- lm(d15N ~ Region, data = groupII)

oneway_N <- Anova(model, Type ="II", white.adjust=TRUE)

oneway_N

#### Group II plots by region ####

# Plot as boxplot

NvsS_C_bp<-ggplot(groupII, aes(Region, d13C)) + geom_boxplot(aes(fill = factor(Region))) +

stat_summary(fun.y=mean, geom="point", shape=5, size=4) +

scale_fill_manual(values = c("#E41A1C","#999999")) +

theme_few(base_size=12) +

scale_y_continuous(breaks = c(0, -5, -10, -15, -20, -25, -30),

labels = c("0","-5.0", "-10.0","-15.0","-20.0","-25.0","-30"),

limits = c(-25,-5)) +

labs(y=expression(paste(delta^{13},"C"," \u0028","\u2030","\u0029"))) + #label the y-axis

theme(legend.position="none") +

stat_summary(fun.data = give.nC, geom = "text", size = 3)

NvsS_C_bp

#ggsave(filename="NvsS_C_bp.pdf", width = 4, height = 4, plot=C_bp)

NvsS_N_bp<-ggplot(groupII, aes(Region, d15N)) + geom_boxplot(aes(fill = factor(Region))) +

stat_summary(fun.y=mean, geom="point", shape=5, size=4) +

scale_fill_manual(values = c("#E41A1C","#999999")) +

theme_few(base_size=12) +

scale_y_continuous(breaks = c(0, 5, 10, 15, 20),

labels = c("0","5.0", "10.0","15.0","20.0"),

limits = c(2,18)) +

labs(y=expression(paste(delta^{15},"N"," \u0028","\u2030","\u0029"))) + #label the y-axis

theme(legend.position="none") +

stat_summary(fun.data = give.nN, geom = "text", size = 3)

NvsS_N_bp

#ggsave(filename="NvsS_N_bp.pdf", width = 4, height = 4, plot=C_bp)

# Plot by Province

# drop the Provinces that have too few data points or no N data

groupII <- filter(groupII, Province != "Hennan")

Province_plot<-ggplot(groupII,aes(d13C,d15N)) +

geom_point(aes(shape = Province, color = Region), size=2) +

scale_shape_manual(values=c(14,13,2,9,3,8,5,6,1,11)) +

scale_color_manual(values=c("#E41A1C","#999999"), labels = c("Loess Plateau", "Yangtze-Huai")) +

scale_y_continuous(breaks = c(0, 5, 10, 15, 20),

labels = c("0","5.0", "10.0","15.0","20.0"),

limits = c(2,18)) +

scale_x_continuous(breaks = c(0, -5, -10, -15, -20, -25),

labels = c("0","-5.0", "-10.0","-15.0","-20.0","-25.0"),

limits = c(-22,-5)) +

labs(x=expression(paste(delta^{13},"C", " \u0028","\u2030","\u0029"))) + #label the x axis

labs(y=expression(paste(delta^{15},"N"," \u0028","\u2030","\u0029"))) + #label the y-axis

theme_few(base_size=12)+

theme(legend.position="right")

Province_plot

#ggsave(filename="Province_plot_groupII.pdf", width = 6, height = 4, plot=Province_plot)

# Combine with boxplots into one figure

GroupII_plots <- plot_grid(NvsS_C_bp,NvsS_N_bp,Province_plot,

ncol = 3,

rel_widths = c(1/4,1/4,1/2),

labels = "auto")

GroupII_plots

#ggsave(filename="GroupII_plots.pdf", width = 10, height = 3, plot=GroupII_plots)

## Compare Provinces

groupII_ProvinceC <- groupII %>%

group_by(Province) %>%

summarise(

Meand13C = mean(d13C),

SDd13C = sd(d13C),

n = n()

)

groupII_ProvinceC

groupII_ProvinceN <- groupII %>%

group_by(Province) %>%

summarise(

Meand15N = mean(d15N, na.rm = TRUE),

SDd15N = sd(d15N, na.rm = TRUE),

n = n()

)

groupII_ProvinceN

## Compare Sites

groupII_Site <- groupII %>%

group_by(Site, Region, Province) %>%

summarise(

Meand15N = mean(d15N),

SDd15N = sd(d15N),

Meand13C = mean(d13C),

SDd13C = sd(d13C),

n = n()

)

groupII_Site

#write_csv(groupII_Site, "groupII_Site.csv")

groupII_Province <- groupII %>%

group_by(Province) %>%

summarise(

Meand15N = mean(d15N),

SDd15N = sd(d15N),

Meand13C = mean(d13C),

SDd13C = sd(d13C),

n = n()

)

Regions <- c("B","B","A","B","A","B","A","A")

groupII_Province <- mutate(groupII_Province, Regions)

#### Group II boxplots ordered N to S ####

groupII$Province<-factor(groupII$Province, levels=c("Inner Mongolia", "Hebei", "Shanxi", "Shandong","Shaanxi","Henan","Jiangsu","Hubei","Zhejiang", "Fujian", "Guangdong"))

# Plot as boxplot

C_groupII_pro<-ggplot(groupII, aes(Province, d13C)) +

geom_boxplot(aes(fill = Region), lwd=0.2, fatten=2) +

scale_fill_manual(values = c("#E41A1C","#999999")) +

theme_few(base_size=12) +

scale_y_continuous(breaks = c(0, -5, -10, -15, -20, -25),

labels = c("0","-5.0", "-10.0","-15.0","-20.0","-25.0"),

limits = c(-22,-5)) +

labs(y=expression(paste(delta^{13},"C"," \u0028","\u2030","\u0029"))) + #label the y-axis

theme(legend.position="none") +

stat_summary(fun.data = give.nC, geom = "text", size = 3)

C_groupII_pro

#ggsave(filename="GroupII_province_C.pdf", width = 8, height = 4, plot=C_groupII_pro)

N_groupII_pro<-ggplot(groupII, aes(Province, d15N)) +

geom_boxplot(aes(fill = Region), lwd=0.2, fatten=2) +

scale_fill_manual(values = c("#E41A1C","#999999")) +

theme_few(base_size=12) +

scale_y_continuous(breaks = c(0, 5, 10, 15, 20),

labels = c("0","5.0", "10.0","15.0","20.0"),

limits = c(2,18)) +

labs(y=expression(paste(delta^{15},"N"," \u0028","\u2030","\u0029"))) + #label the y-axis

theme(legend.position="none") +

stat_summary(fun.data = give.nN, geom = "text", size = 3)

N_groupII_pro

#ggsave(filename="GroupII_province.pdf", width = 8, height = 4, plot=N_groupII_pro)

# Combine with boxplots into one figure

groupII_province <- plot_grid(C_groupII_pro, N_groupII_pro,

ncol = 1,

labels = "auto")

groupII_province

#ggsave(filename="GroupII_province_NS.pdf", width = 8, height = 5, plot=groupII_province)

#### Parametric Stats; group II provinces ####

# Are Provinces different?

fit_C <- aov(d13C ~ Province, data = groupII)

fit_C

summary(fit_C)

hist(fit_C$residuals)

library(car)

leveneTest(d13C ~ Province, data = groupII)

model <- lm(d13C ~ Province, data = groupII)

oneway_C <- Anova(model, Type ="II", white.adjust=TRUE)

oneway_C

TukeyHSD(fit_C)

cld_C <- HSD.test(fit_C, "Province", group=TRUE)

cld_C

fit_N <- aov(d15N ~ Province, data = groupII)

fit_N

summary(fit_N)

hist(fit_N$residuals)

leveneTest(d15N ~ Province, data = groupII)

model <- lm(d15N ~ Province, data = groupII)

oneway_N <- Anova(model, Type ="II", white.adjust=TRUE)

oneway_N

TukeyHSD(fit_N)

cld_N <- HSD.test(fit_N, "Province", group=TRUE)

cld_N

#####################

##### Group III #####

#####################

## Compare Regions (A and C) after 2000BC

groupIII <- all %>%

filter(Time_Group == "III", Region != "B")

# Plot colored by region

Plot3<-ggplot(groupIII,aes(d13C,d15N)) +

geom_point(aes(color=Region), size=3, shape=21) +

scale_color_brewer(palette="Set1") +

scale_y_continuous(breaks = c(0, 5, 10, 15, 20),

labels = c("0","5.0", "10.0","15.0","20.0"),

limits = c(2,18)) +

scale_x_continuous(breaks = c(0, -5, -10, -15, -20, -25),

labels = c("0","-5.0", "-10.0","-15.0","-20.0","-25.0"),

limits = c(-22,-5)) +

labs(x=expression(paste(delta^{13},"C", " \u0028","\u2030","\u0029"))) + #label the x axis

labs(y=expression(paste(delta^{15},"N"," \u0028","\u2030","\u0029"))) + #label the y-axis

labs(title="Loess Plateau vs. Continental interior, < 2000 BC") +

theme_few(base_size=12) +

theme(legend.position="right")

Plot3

#ggsave(filename="GroupIII.pdf", width = 6, height = 4, plot=Plot3)

#### Parametric Stats; group III regions #####

# Are regions significantly different?

fit_C <- aov(d13C ~ Region, data = groupIII)

fit_C

summary(fit_C)

hist(fit_C$residuals)

leveneTest(d13C ~ Region, data = groupIII)

model <- lm(d13C ~ Region, data = groupIII)

oneway_C <- Anova(model, Type ="II", white.adjust=TRUE)

oneway_C

fit_N <- aov(d15N ~ Region, data = groupIII)

fit_N

summary(fit_N)

hist(fit_N$residuals)

leveneTest(d15N ~ Region, data = groupIII)

model <- lm(d15N ~ Region, data = groupIII)

oneway_N <- Anova(model, Type ="II", white.adjust=TRUE)

oneway_N

#### Group III plots by region ####

# Plot as boxplot

C_bp_groupIII<-ggplot(groupIII, aes(Region, d13C)) + geom_boxplot(aes(fill = factor(Region))) +

stat_summary(fun.y=mean, geom="point", shape=5, size=4) +

scale_fill_manual(values = c("#E41A1C","#377eb8")) +

theme_few(base_size=12) +

scale_y_continuous(breaks = c(0, -5, -10, -15, -20, -25),

labels = c("0","-5.0", "-10.0","-15.0","-20.0","-25.0"),

limits = c(-22,-5)) +

labs(y=expression(paste(delta^{13},"C"," \u0028","\u2030","\u0029"))) + #label the y-axis

theme(legend.position="none") +

stat_summary(fun.data = give.nC, geom = "text", size = 3)

C_bp_groupIII

#ggsave(filename="GroupIII_C_bp", width = 4, height = 4, plot=C_bp_groupIII)

N_bp_groupIII<-ggplot(groupIII, aes(Region, d15N)) + geom_boxplot(aes(fill = factor(Region))) +

stat_summary(fun.y=mean, geom="point", shape=5, size=4) +

scale_fill_manual(values = c("#E41A1C","#377eb8")) +

theme_few(base_size=12) +

scale_y_continuous(breaks = c(0, 5, 10, 15, 20),

labels = c("0","5.0", "10.0","15.0","20.0"),

limits = c(2,18)) +

labs(y=expression(paste(delta^{15},"N"," \u0028","\u2030","\u0029"))) + #label the y-axis

theme(legend.position="none") +

stat_summary(fun.data = give.nN, geom = "text", size = 3)

N_bp_groupIII

#ggsave(filename="GroupIII_N_bp.pdf", width = 4, height = 4, plot=N_bp_groupIII)

## Compare Provinces

groupIII_ProvinceC <- groupIII %>%

group_by(Province) %>%

summarise(

Meand13C = mean(d13C, na.rm=TRUE),

SDd13C = sd(d13C, na.rm=TRUE),

n = n()

)

groupIII_ProvinceC

groupIII_ProvinceN <- groupIII %>%

group_by(Province) %>%

summarise(

Meand15N = mean(d15N, na.rm = TRUE),

SDd15N = sd(d15N, na.rm = TRUE),

n = n()

)

groupIII_ProvinceN

## Compare Sites

groupIII_Site <- groupIII %>%

group_by(Site, Region, Province, Reference) %>%

summarise(

Meand15N = mean(d15N),

SDd15N = sd(d15N),

Meand13C = mean(d13C),

SDd13C = sd(d13C),

n = n()

)

groupIII_Site

#write_csv(groupIII_Site, "groupIII_Site.csv")

# drop the Provinces that have too few data points

groupIII <- groupIII %>%

filter(Province != "")

groupIII_Province <- groupIII %>%

group_by(Province) %>%

summarise(

Meand15N = mean(d15N, na.rm = TRUE),

SDd15N = sd(d15N, na.rm = TRUE),

Meand13C = mean(d13C, na.rm = TRUE),

SDd13C = sd(d13C, na.rm = TRUE),

n = n()

)

Regions <- c("C","A","A","C","C","A","A","A","C")

groupIII_Province <- mutate(groupIII_Province, Regions)

# Scatter plot by province

Province_plot3<-ggplot(groupIII,aes(d13C,d15N)) +

geom_point(aes(shape=Province, color=Region), size=2) +

scale_shape_manual(values=c(7,0,2,3,4,5,6,1,10)) +

scale_color_manual(values = c("#E41A1C","#377eb8"), labels = c("Loess Plateau", "CI")) +

scale_y_continuous(breaks = c(0, 5, 10, 15, 20),

labels = c("0","5.0", "10.0","15.0","20.0"),

limits = c(2,18)) +

scale_x_continuous(breaks = c(0, -5, -10, -15, -20, -25),

labels = c("0","-5.0", "-10.0","-15.0","-20.0","-25.0"),

limits = c(-22,-5)) +

labs(x=expression(paste(delta^{13},"C", " \u0028","\u2030","\u0029"))) + #label the x axis

labs(y=expression(paste(delta^{15},"N"," \u0028","\u2030","\u0029"))) + #label the y-axis

theme_few(base_size=12)+

theme(legend.position="right")

Province_plot3

#ggsave(filename="GroupIII_Province_plot.pdf", width = 6, height = 4, plot=Province_plot3)

# Combine with boxplots into one figure

GroupIII_plots <- plot_grid(C_bp_groupIII,N_bp_groupIII,Province_plot3,

ncol = 3,

rel_widths = c(1/4,1/4,1/2),

labels = "auto")

GroupIII_plots

#ggsave(filename="GroupIII_plots.pdf", width = 10, height = 3, plot=GroupIII_plots)

#### Group III boxplots ordered E to W ####

groupIII$Province<-factor(groupIII$Province, levels=c("Shandong", "Hebei", "Shanxi", "Henan","Shaanxi","Inner Mongolia","Gansu","Qinghai","Xinjiang"))

C_groupIII_pro<-ggplot(groupIII, aes(Province, d13C)) +

geom_boxplot(aes(fill = Region), lwd=0.2, fatten=2) +

scale_fill_manual(values = c("#E41A1C","#377eb8")) +

theme_few(base_size=12) +

scale_y_continuous(breaks = c(0, -5, -10, -15, -20, -25),

labels = c("0","-5.0", "-10.0","-15.0","-20.0","-25.0"),

limits = c(-22,-5)) +

labs(y=expression(paste(delta^{13},"C"," \u0028","\u2030","\u0029"))) + #label the y-axis

theme(legend.position="none") +

stat_summary(fun.data = give.nC, geom = "text", size = 3)

C_groupIII_pro

#ggsave(filename="GroupIII_province_C.pdf", width = 8, height = 4, plot=C_groupIII_pro)

N_groupIII_pro<-ggplot(groupIII, aes(Province, d15N)) +

geom_boxplot(aes(fill = Region), lwd=0.2, fatten=2) +

scale_fill_manual(values = c("#E41A1C","#377eb8")) +

theme_few(base_size=12) +

scale_y_continuous(breaks = c(0, 5, 10, 15, 20),

labels = c("0","5.0", "10.0","15.0","20.0"),

limits = c(2,18)) +

labs(y=expression(paste(delta^{15},"N"," \u0028","\u2030","\u0029"))) + #label the y-axis

theme(legend.position="none") +

stat_summary(fun.data = give.nN, geom = "text", size = 3)

N_groupIII_pro

#ggsave(filename="GroupIII_province_N.pdf", width = 8, height = 4, plot=N_groupIII_pro)

# Combine with boxplots into one figure

groupIII_province <- plot_grid(C_groupIII_pro, N_groupIII_pro,

ncol = 1,

labels = "auto")

groupIII_province

#ggsave(filename="GroupIII_province_EW.pdf", width = 8, height = 5, plot=groupIII_province)

#### Parametric stats; group III provinces #####

# Are Provinces different?

fit_C <- aov(d13C ~ Province, data = groupIII)

fit_C

summary(fit_C)

hist(fit_C$residuals)

leveneTest(d13C ~ Province, data = groupIII)

model <- lm(d13C ~ Province, data = groupIII)

oneway_C <- Anova(model, Type ="II", white.adjust=TRUE)

oneway_C

TukeyHSD(fit_C)

cld_C <- HSD.test(fit_C, "Province", group=TRUE)

cld_C$groups

fit_N <- aov(d15N ~ Province, data = groupIII)

fit_N

summary(fit_N)

hist(fit_N$residuals)

leveneTest(d15N ~ Province, data = groupIII)

model <- lm(d15N ~ Province, data = groupIII)

oneway_N <- Anova(model, Type ="II", white.adjust=TRUE)

oneway_N

TukeyHSD(fit_N)

cld_N <- HSD.test(fit_N, "Province", group=TRUE)

cld_N

###########################

##### Sex Differences #####

###########################

# break into groups by time bin

sex <- filter(all, Sex == "M" | Sex == "F")

sex_I <- filter(sex, Time_Group == "I")

sex_II <- filter(sex, Time_Group == "II")

sex_III <- filter(sex, Time_Group == "III")

# plot by region and time group

sexI_plot <- ggplot(sex_I,aes(d13C,d15N)) +

geom_point(aes(color = Region, shape = Sex), size=3, stroke=0.5) +

scale_shape_manual(values=c(1,3)) +

scale_color_manual(values = c("#E41A1C","#377eb8","#999999"), labels = c("Loess Plateau", "Yangtze-Huai", "Continental interior")) +

scale_y_continuous(breaks = c(0, 5, 10, 15, 20),

labels = c("0","5.0", "10.0","15.0","20.0"),

limits = c(2,18)) +

scale_x_continuous(breaks = c(0, -5, -10, -15, -20, -25),

labels = c("0","-5.0", "-10.0","-15.0","-20.0","-25.0"),

limits = c(-22,-5)) +

labs(x=expression(paste(delta^{13},"C", " \u0028","\u2030","\u0029"))) + #label the x axis

labs(y=expression(paste(delta^{15},"N"," \u0028","\u2030","\u0029"))) + #label the y-axis

theme_few(base_size=12)

sexI_plot

#ggsave(filename="SexI.pdf", width = 6, height = 4, plot=sexI_plot)

#### Group II Sex Differences ####

sexII_plot <- ggplot(sex_II,aes(d13C,d15N)) +

geom_point(aes(color = Region, shape = Sex, alpha = Sex), size=3, stroke=0.5) +

scale_color_manual(values = c("#E41A1C","#999999","#377eb8"), labels = c("Loess Plateau", "Yangtze-Huai", "Continental interior")) +

scale_shape_manual(values=c(2,1), labels = c("Female","Male")) +

scale_alpha_discrete(range = c(0.35, 0.9)) +

scale_y_continuous(breaks = c(0, 5, 10, 15, 20),

labels = c("0","5.0", "10.0","15.0","20.0"),

limits = c(2,18)) +

scale_x_continuous(breaks = c(0, -5, -10, -15, -20, -25),

labels = c("0","-5.0", "-10.0","-15.0","-20.0","-25.0"),

limits = c(-22,-5)) +

labs(x=expression(paste(delta^{13},"C", " \u0028","\u2030","\u0029"))) + #label the x axis

labs(y=expression(paste(delta^{15},"N"," \u0028","\u2030","\u0029"))) + #label the y-axis

theme_few(base_size=12) +

guides(alpha=FALSE)

sexII_plot

#ggsave(filename="SexII.pdf", width = 6, height = 4, plot=sexII_plot)

groupII_Sex <- sex_II %>%

group_by(Region, Sex) %>%

summarise(

Meand15N = mean(d15N),

SDd15N = sd(d15N),

Meand13C = mean(d13C),

SDd13C = sd(d13C),

n = n()

)

groupII_Sex

# Group II boxplots by sex

C_bp_sexII<-ggplot(sex_II, aes(x = Region, y = d13C, fill = Region, alpha = Sex)) +

geom_boxplot(aes(fill = Region, alpha = Sex), fatten=2, lwd=0.2) +

stat_summary(fun.y=mean, geom="point", shape=5, size=2, position = position_dodge(width=0.75)) +

scale_fill_manual(values = c("#E41A1C","#999999","#377eb8"), labels = c("Loess Plateau", "Yangtze-Huai", "Continental interior")) +

scale_alpha_discrete(range = c(0.35, 0.9), labels = c("female", "male")) +

scale_y_continuous(breaks = c(0, -5, -10, -15, -20, -25),

labels = c("0","-5.0", "-10.0","-15.0","-20.0","-25.0"),

limits = c(-22,-5)) +

theme_few(base_size=12) +

labs(y=expression(paste(delta^{13},"C"," \u0028","\u2030","\u0029"))) + #label the y-axis

theme(legend.position="none") +

stat_summary(fun.data = "give.nC", geom = "text", position = position_dodge(width=0.8), size = 2.5)

C_bp_sexII

N_bp_sexII<-ggplot(sex_II, aes(x = Region, y = d15N, fill = Region, alpha = Sex)) +

geom_boxplot(aes(fill = Region, alpha = Sex), lwd=0.2, fatten=2) +

stat_summary(fun.y=mean, geom="point", shape=5, size=2, position = position_dodge(width=0.75)) +

scale_fill_manual(values = c("#E41A1C","#999999", "#377eb8"), labels = c("Loess Plateau", "Yangtze-Huai", "Continental interior")) +

scale_alpha_discrete(range = c(0.35, 0.9), labels = c("female", "male")) +

scale_y_continuous(breaks = c(0, 5, 10, 15, 20),

labels = c("0","5.0", "10.0","15.0","20.0"),

limits = c(2,18)) +

theme_few(base_size=12) +

labs(y=expression(paste(delta^{15},"N"," \u0028","\u2030","\u0029"))) + #label the y-axis

theme(legend.position="none") +

stat_summary(fun.data = "give.nN", geom = "text", position = position_dodge(width=0.8), size = 2.5)

N_bp_sexII

#plot together

sexII_plots <- plot_grid(C_bp_sexII, N_bp_sexII, sexII_plot,

ncol = 3,

rel_widths = c(1/4,1/4,1/2),

labels = "auto")

sexII_plots

#ggsave(filename="II_region.pdf", width = 10, height = 3, plot=sexII_plots)

#### Parametric stats; Group II sex and region ####

# Are there significant differences by sex?

fit_C <- aov(d13C ~ Sex, data = sex_II)

fit_C

summary(fit_C)

hist(fit_C$residuals)

leveneTest(d13C ~ Sex, data = sex_II)

fit_N <- aov(d15N ~ Sex, data = sex_II)

fit_N

summary(fit_N)

hist(fit_N$residuals)

leveneTest(d15N ~ Sex, data = sex_II)

# Are there significant differences by sex and region?

fit_C <- aov(d13C ~ Region * Sex, data = sex_II)

fit_C

summary(fit_C)

hist(fit_C$residuals)

leveneTest(d13C ~ Region * Sex, data = sex_II)

TukeyHSD(fit_C)

lsmeans = lsmeans::lsmeans ### Uses the lsmeans function from the lsmeans package

leastsquare <- lsmeans(fit_C,

pairwise ~ Region:Sex,

adjust="tukey")

cld(leastsquare[[1]],

alpha=.05,

Letters=letters)

fit_N <- aov(d15N ~ Region * Sex, data = sex_II)

fit_N

summary(fit_N)

hist(fit_N$residuals)

leveneTest(d15N ~ Region * Sex, data = sex_II)

TukeyHSD(fit_N)

lsmeans = lsmeans::lsmeans ### Uses the lsmeans function from the lsmeans package

leastsquare <- lsmeans(fit_N,

pairwise ~ Region:Sex,

adjust="tukey")

cld(leastsquare[[1]],

alpha=.05,

Letters=letters)

#### Group III Sex Differences ####

groupIII_Sex <- sex_III %>%

filter(!is.na(d15N)) %>%

filter(!is.na(d13C)) %>%

group_by(Region, Sex) %>%

summarise(

Mediand15N = median(d15N),

Meand15N = mean(d15N),

SDd15N = sd(d15N),

Mediand13C = median(d13C),

Meand13C = mean(d13C),

SDd13C = sd(d13C),

n = n()

)

groupIII_Sex

sexIII_plot <- ggplot(sex_III,aes(d13C,d15N)) +

geom_point(aes(color = Region, alpha = Sex, shape = Sex), size=2, stroke=0.5) +

scale_color_manual(values = c("#E41A1C","#377eb8","#999999"), labels = c("Loess Plateau", "Yangtze-Huai", "CI")) +

scale_shape_manual(values=c(2,1)) +

scale_alpha_discrete(range = c(0.35, 0.9), labels = c("female", "male")) +

scale_y_continuous(breaks = c(0, 5, 10, 15, 20),

labels = c("0","5.0", "10.0","15.0","20.0"),

limits = c(2,18)) +

scale_x_continuous(breaks = c(0, -5, -10, -15, -20, -25),

labels = c("0","-5.0", "-10.0","-15.0","-20.0","-25.0"),

limits = c(-22,-5)) +

labs(x=expression(paste(delta^{13},"C", " \u0028","\u2030","\u0029"))) + #label the x axis

labs(y=expression(paste(delta^{15},"N"," \u0028","\u2030","\u0029"))) + #label the y-axis

theme_few(base_size=12) +

guides(alpha = FALSE)

sexIII_plot

#ggsave(filename="SexIII.pdf", width = 6, height = 4, plot=sexIII_plot)

#boxplots for C and N by Region within III

C_bp_sexIII<-ggplot(sex_III, aes(x = Region, y = d13C, fill = Region, alpha = Sex)) +

geom_boxplot(aes(fill = Region, alpha = Sex), fatten=2, lwd=0.2) +

stat_summary(fun.y=mean, geom="point", shape=5, size=2, position = position_dodge(width=0.75)) +

scale_fill_manual(values = c("#E41A1C","#377eb8","#999999"), labels = c("Loess Plateau", "Yangtze-Huai", "Continental interior")) +

scale_alpha_discrete(range = c(0.35, 0.9), labels = c("female", "male")) +

scale_y_continuous(breaks = c(0, -5, -10, -15, -20, -25),

labels = c("0","-5.0", "-10.0","-15.0","-20.0","-25.0"),

limits = c(-22,-5)) +

theme_few(base_size=12) +

labs(y=expression(paste(delta^{13},"C"," \u0028","\u2030","\u0029"))) + #label the y-axis

theme(legend.position="none") +

stat_summary(fun.data = "give.nC", geom = "text", position = position_dodge(width=0.8), size = 3)

C_bp_sexIII

N_bp_sexIII<-ggplot(sex_III, aes(x = Region, y = d15N, fill = Region, alpha = Sex)) +

geom_boxplot(aes(fill = Region, alpha = Sex), lwd=0.2, fatten=2) +

stat_summary(fun.y=mean, geom="point", shape=5, size=2, position = position_dodge(width=0.75)) +

scale_fill_manual(values = c("#E41A1C","#377eb8","#999999"), labels = c("Loess Plateau", "Yangtze-Huai", "Continental interior")) +

scale_alpha_discrete(range = c(0.35, 0.9), labels = c("female", "male")) +

scale_y_continuous(breaks = c(0, 5, 10, 15, 20),

labels = c("0","5.0", "10.0","15.0","20.0"),

limits = c(2,18)) +

theme_few(base_size=12) +

labs(y=expression(paste(delta^{15},"N"," \u0028","\u2030","\u0029"))) + #label the y-axis

theme(legend.position="none") +

stat_summary(fun.data = "give.nN", geom = "text", position = position_dodge(width=0.8), size = 3)

N_bp_sexIII

# plot together

sexIII_plots <- plot_grid(C_bp_sexIII, N_bp_sexIII, sexIII_plot,

ncol = 3,

rel_widths = c(1/4,1/4,1/2),

labels = "auto")

sexIII_plots

#ggsave(filename="III_region_sex.pdf", width = 10, height = 3, plot=sexIII_plots)

#### Parametric stats; Group III sex and region ####

# Are there significant differences by sex and region?

fit_C <- aov(d13C ~ Region * Sex, data = sex_III)

fit_C

summary(fit_C)

hist(fit_C$residuals)

leveneTest(d13C ~ Region * Sex, data = sex_III)

TukeyHSD(fit_C)

lsmeans = lsmeans::lsmeans ### Uses the lsmeans function from the lsmeans package

leastsquare <- lsmeans(fit_C,

pairwise ~ Region:Sex,

adjust="tukey")

cld(leastsquare[[1]],

alpha=.05,

Letters=letters)

fit_N <-aov(d15N ~ Region * Sex, data = sex_III)

fit_N

summary(fit_N)

hist(fit_N$residuals)

shapiro.test(fit_N$residuals)

leveneTest(d15N ~ Region * Sex, data = sex_III)

TukeyHSD(fit_N)

lsmeans = lsmeans::lsmeans ### Uses the lsmeans function from the lsmeans package

leastsquare <- lsmeans(fit_N,

pairwise ~ Region:Sex,

adjust="tukey")

cld(leastsquare[[1]],

alpha=.05,

Letters=letters)

#### Sex differences by Province - Group II ####

# all regions

# boxplots ordered N to S

sex_II <- filter(sex_II, Province != "Hennan") # drop the Provinces that have too few data points or no N data

sex_II$Province<-factor(sex_II$Province, levels=c("Shanxi", "Shandong","Shaanxi","Henan","Fujian"))

groupII_Sex_province <- sex_II %>%

filter(!is.na(d15N)) %>%

filter(!is.na(d13C)) %>%

group_by(Province, Region,Sex) %>%

summarise(

Mediand15N = median(d15N),

Meand15N = mean(d15N),

SDd15N = sd(d15N),

Mediand13C = median(d13C),

Meand13C = mean(d13C),

SDd13C = sd(d13C),

n = n()

)

groupII_Sex_province

C_groupII_NS_sex<-ggplot(sex_II, aes(x = Province, y = d13C, fill = Region, alpha = Sex)) +

geom_boxplot(aes(fill = Region, alpha = Sex), lwd=0.2, fatten=2) +

stat_summary(fun.y=mean, geom="point", shape=5, size=1, position = position_dodge(width=0.75)) +

scale_fill_manual(values = c("#E41A1C","#999999")) +

scale_alpha_discrete(range = c(0.35, 0.9), labels = c("female", "male")) +

theme_few(base_size=12) +

ylim(-25,-5) +

labs(y=expression(paste(delta^{13},"C"," \u0028","\u2030","\u0029"))) + #label the y-axis

theme(legend.position="none") +

stat_summary(fun.data = "give.nC", geom = "text", position = position_dodge(width=0.8), size = 3)

C_groupII_NS_sex

#ggsave(filename="GroupII_province_C_sex.pdf", width = 8, height = 4, plot=C_groupII_pro_sex)

N_groupII_NS_sex<-ggplot(sex_II, aes(x = Province, y = d15N, fill = Region, alpha = Sex)) +

geom_boxplot(aes(fill = Region, alpha = Sex), lwd=0.2, fatten=2) +

stat_summary(fun.y=mean, geom="point", shape=5, size=1, position = position_dodge(width=0.75)) +

scale_fill_manual(values = c("#E41A1C","#999999")) +

scale_alpha_discrete(range = c(0.35, 0.9), labels = c("female", "male")) +

theme_few(base_size=12) +

ylim(2,18) +

labs(y=expression(paste(delta^{15},"N"," \u0028","\u2030","\u0029"))) + #label the y-axis

theme(legend.position="none") +

stat_summary(fun.data = "give.nN", geom = "text", position = position_dodge(width=0.8), size = 3)

N_groupII_NS_sex

#ggsave(filename="GroupII_province_N_sex.pdf", width = 8, height = 4, plot=N_groupII_pro_sex)

# Combine with boxplots into one figure

groupII_sex_NS <- plot_grid(C_groupII_NS_sex, N_groupII_NS_sex,

ncol = 1,

labels = "auto")

groupII_sex_NS

#ggsave(filename="groupII_sex.pdf", width = 6, height = 4, plot=groupII_sex_NS)

## Test for significant differences among groups using anova

fit_C <- aov(d13C ~ Sex*Province*Region, data = sex_II)

fit_C

summary(fit_C)

TukeyHSD(fit_C)

lsmeans = lsmeans::lsmeans ### Uses the lsmeans function from the lsmeans package

leastsquare <- lsmeans(fit_C,

pairwise ~ Province:Sex:Region,

adjust="tukey")

cld(leastsquare[[1]],

alpha=.05,

Letters=letters)

fit_N <- aov(d15N ~ Sex*Province*Region, data = sex_II)

fit_N

summary(fit_N)

TukeyHSD(fit_N)

leastsquare <- lsmeans(fit_N,

pairwise ~ Province:Sex:Region,

adjust="tukey")

cld(leastsquare[[1]],

alpha=.05,

Letters=letters)

#### Sex Differences by Province - Group III ####

# boxplots ordered E to W

sex_III <- filter(sex_III, Province != "Hennan" & Province != "Qinghai")

sex_III$Province<-factor(sex_III$Province, levels=c("Anhui","Yunnan","Shandong", "Hebei", "Shanxi", "Henan","Shaanxi","Inner Mongolia","Gansu","Xinjiang"))

C_groupIII_EW_sex<-ggplot(sex_III, aes(x = Province, y = d13C, fill = Region, alpha = Sex)) +

geom_boxplot(aes(fill = Region, alpha = Sex), lwd=0.2, fatten=2) +

stat_summary(fun.y=mean, geom="point", shape=5, size=1, position = position_dodge(width=0.75)) +

scale_fill_manual(values = c("#E41A1C","#377eb8","#999999")) +

scale_alpha_discrete(range = c(0.35, 0.9), labels = c("female", "male")) +

theme_few(base_size=12) +

ylim(-25,-5) +

labs(y=expression(paste(delta^{13},"C"," \u0028","\u2030","\u0029"))) + #label the y-axis

theme(legend.position="none") +

stat_summary(fun.data = "give.nC", geom = "text", position = position_dodge(width=0.8), size = 2.5)

C_groupIII_EW_sex

#ggsave(filename="GroupIII_EW_C_sex.pdf", width = 8, height = 4, plot=C_groupIII_EW_sex)

N_groupIII_EW_sex<-ggplot(sex_III, aes(x = Province, y = d15N, fill = Region, alpha = Sex)) +

geom_boxplot(aes(fill = Region, alpha = Sex), lwd=0.2, fatten=2) +

stat_summary(fun.y=mean, geom="point", shape=5, size=1, position = position_dodge(width=0.75)) +

scale_fill_manual(values = c("#E41A1C","#377eb8", "#999999")) +

scale_alpha_discrete(range = c(0.35, 0.9), labels = c("female", "male")) +

theme_few(base_size=12) +

ylim(2,18) +

labs(y=expression(paste(delta^{15},"N"," \u0028","\u2030","\u0029"))) + #label the y-axis

theme(legend.position="none") +

stat_summary(fun.data = "give.nN", geom = "text", position = position_dodge(width=0.8), size = 2.5)

N_groupIII_EW_sex

#ggsave(filename="GroupIII_province_N_sex.pdf", width = 8, height = 4, plot=N_groupIII_EW_sex)

# Combine with boxplots into one figure

groupIII_sex_EW <- plot_grid(C_groupIII_EW_sex, N_groupIII_EW_sex,

ncol = 1,

labels = "auto")

groupIII_sex_EW

#ggsave(filename="groupIII_sex_EW.pdf", width = 8.5, height = 8, plot=groupIII_sex_EW)

groupIII_Sex_province <- sex_III %>%

filter(!is.na(d15N)) %>%

filter(!is.na(d13C)) %>%

group_by(Province, Region,Sex) %>%

summarise(

Mediand15N = median(d15N),

Meand15N = mean(d15N),

SDd15N = sd(d15N),

Mediand13C = median(d13C),

Meand13C = mean(d13C),

SDd13C = sd(d13C),

n = n()

)

groupIII_Sex_province

fit_C <- aov(d13C ~ Province * Sex, data = sex_III)

fit_C

summary(fit_C)

hist(fit_C$residuals)

leveneTest(d13C ~ Province * Sex, data = sex_III)

TukeyHSD(fit_C)

lsmeans = lsmeans::lsmeans ### Uses the lsmeans function from the lsmeans package

leastsquare <- lsmeans(fit_C,

pairwise ~ Province:Sex,

adjust="tukey")

cld(leastsquare[[1]],

alpha=.05,

Letters=letters)

fit_N <- aov(d15N ~ Province * Sex, data = sex_III)

fit_N

summary(fit_N)

hist(fit_N$residuals)

leveneTest(d15N ~ Province * Sex, data = sex_III)

TukeyHSD(fit_N)

lsmeans = lsmeans::lsmeans ### Uses the lsmeans function from the lsmeans package

leastsquare <- lsmeans(fit_N,

pairwise ~ Province:Sex,

adjust="tukey")

cld(leastsquare[[1]],

alpha=.05,

Letters=letters)
